# From tumor mutational burden to characteristic targets analysis: Identifying the predictive biomarkers and natural product interventions in cancer management

**DOI:** 10.3389/fnut.2022.989989

**Published:** 2022-09-20

**Authors:** Cun Liu, Yang Yu, Ge Wang, Jingyang Liu, Ruijuan Liu, Lijuan Liu, Xiaoxu Yang, Huayao Li, Chundi Gao, Yi Lu, Jing Zhuang

**Affiliations:** ^1^College of Traditional Chinese Medicine, Weifang Medical University, Weifang, China; ^2^College of Traditional Chinese Medicine, Shandong University of Traditional Chinese Medicine, Jinan, China; ^3^Clinical Medical Colleges, Weifang Medical University, Weifang, China; ^4^First School of Clinical Medicine, Shandong University of Traditional Chinese Medicine, Jinan, China; ^5^Department of Oncology, Weifang Traditional Chinese Hospital, Weifang, China; ^6^Department of Special Medicine, School of Basic Medicine, Qingdao University, Qingdao, China; ^7^School of Life Sciences and Technology, Weifang Medical University, Weifang, China; ^8^Department of Clinical Nutrition, The Cancer Hospital of the University of Chinese Academy of Sciences (Zhejiang Cancer Hospital), Institute of Basic Medicine and Cancer, Chinese Academy of Sciences, Hangzhou, China; ^9^Key Laboratory of Traditional Chinese Medicine Oncology, Zhejiang Cancer Hospital, Hangzhou, China

**Keywords:** next-generation sequencing, tumor mutation burden, targeted therapy and immunotherapy, *LRP1B*, *APC*

## Abstract

High-throughput next-generation sequencing (NGS) provides insights into genome-wide mutations and can be used to identify biomarkers for the prediction of immune and targeted responses. A deeper understanding of the molecular biological significance of genetic variation and effective interventions is required and ultimately needs to be associated with clinical benefits. We conducted a retrospective observational study of patients in two cancer cohorts who underwent NGS in a “real-world” setting. The association between differences in tumor mutational burden (TMB) and clinical presentation was evaluated. We aimed to identify several key mutation targets and describe their biological characteristics and potential clinical value. A pan-cancer dataset was downloaded as a verification set for further analysis and summary. Natural product screening for the targeted intervention of key markers was also achieved. The majority of tumor patients were younger adult males with advanced cancer. The gene identified with the highest mutation rate was *TP53*, followed by *PIK3CA*, *EGFR*, and *LRP1B*. The association of TMB (0–103.7 muts/Mb) with various clinical subgroups was determined. More frequent mutations, such as in *LRP1B*, as well as higher levels of ferritin and neuron-specific enolase, led to higher TMB levels. Further analysis of the key targets, *LRP1B* and *APC*, was performed, and mutations in *LRP1B* led to better immune benefits compared to *APC*. *APC*, one of the most frequently mutated genes in gastrointestinal tumors, was further investigated, and the potential interventions by cochinchinone B and rottlerin were clarified. In summary, based on the analysis of the characteristics of gene mutations in the “real world,” we obtained the potential association indicators of TMB, found the key signatures *LRP1B* and *APC*, and further described their biological significance and potential interventions.

## Background

Despite the rapid development of various clinical management strategies, cancer remains a dominant threat to human health in the 21st century (1). This is partly due to the fact that cancer therapies are still “one-size-fits-all” models based on organ-centric approaches that often fail to consider the personalized genomic landscape of tumors. Personalized medicine, which is designed to match the right drugs to the individual patients, is an attractive strategy that promises to improve efficacy while reducing side effects through the integration of genomic, transcriptomic, and proteomic data from tumor samples in oncology decision-making (2). Cancer treatment has benefited from advances in precision medicine, making it more relevant and effective. This is particularly true for targeted therapy models against driver genomic alterations, which have improved outcomes for patients with different types of cancer. This approach heavily relies on the efforts of the scientific community over the past few decades to define the cancer genomic landscape, thereby laying the foundation for personalized medicine and improving our understanding of cancer biology and tumor diversity (3); the development of next-generation sequencing (NGS) that has allowed rapid identification of comprehensive tumor genome profiles and their mutations (base pair substitution, copy number variation, insertion/deletion, and rearrangement), which can be used to match patients with targeted therapies against these carcinogenic drivers (4). Although genomics seems to be the most relevant starting point for the precision medicine approach, determining the molecular phenotype and mutation characteristics is necessary to better understand tumors and improve the overall efficacy of precision medicine approaches in the clinical management of cancer patients.

NGS is a high-throughput sequencing method that can simultaneously identify millions of data points; furthermore, it can provide a static measure of changes within the tumor genome, many of which are known to influence the tumor’s response to specific clinical interventions (5). In 2017, the FDA approved FoundationOne CDx, the first extensive concomitant diagnostic tool for solid tumors (6). Since then, NGS panels based on various gene sizes have been widely evaluated. NGS has successfully guided the clinical choice of targeted therapies and immunotherapy for various cancers through the identification of several actionable variants, EGFR, HER2, and PARP, achieving significant clinical benefits in multiple cancer types (7–9). Furthermore, ongoing studies and clinical trials have begun to evaluate tumor mutational burden (TMB) and microsatellite instability (MSI). High-throughput analysis methods are constantly being developed in parallel with sophisticated data analysis software tools to use these parameters to improve therapeutic efficacy (10).

To date, several studies have shown that molecular genomic profiling is a key tool for identifying clinically relevant information for the development of effective personalized therapeutic interventions (11). Because of its success, this approach has remained a strong focus in anticancer exploration. The development of personalized medicine, led by NGS, combines the hot topics of cancer management and genomics, and the number of cancer genomes sequenced continues to grow exponentially; however, a relatively small proportion of cases have been treated using molecular-guided interventions. Therefore, further work identifying the potential benefits and optimal use of targeted therapy or immunotherapy is needed to design more relevant clinical evaluations (12).

Our study retrospectively analyzed the “real-world” evidence to determine the comprehensive genomic profile and the impact of the routine inclusion of NGS. We analyzed the benefits of NGS-guidance to patients and their relevant clinical characteristics. Based on a high-throughput database and a large sample immunotherapy cohort, the immunological characteristics of the key target, *LRP1B*, were explored. Further, molecular docking analysis of APC was performed using a small molecule library, and the intervention value of cochinchinone B and rottlerin on the *APC* gene was determined. As the *APC* gene is one of the most frequently mutated known drivers in colorectal cancer, our study provides potential intervention measures for the associated disease.

## Materials and methods

### Study design and patients

**FIGURE 1:**
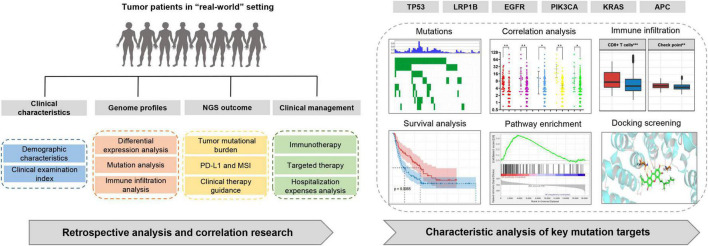
Study design. Retrospective analysis of tumor patients in a real-world setting and association analysis of clinical and mutation targets characteristics.

All NGS-based genomic profiling assays were commercially available multi-gene panels produced by Yikon Genomics (Shanghai, China). We conducted a retrospective, observational study of cancer patients who underwent NGS, and the design of this study is described in Figure 1. These patients underwent comprehensive genomic profiling (CGP) of tumor or body fluid samples, following the requisite consent protocols. The patients’ CGP, gene mutation, TMB, and MSI expression were determined; their clinical and treatment information was extracted from the electronic medical records of the Weifang Medical System. Patients previously treated with targeted drugs were excluded because there was some concern that this may influence their mutational profile. This study was conducted in accordance with the Declaration of Helsinki, and the protocol was approved by the ethics committee of Weifang Traditional Chinese Hospital.

### Library construction and next-generation sequencing

Each sample underwent genomic extraction, and 30–500 ng of this DNA was used to generate fragments of approximately 150 bp for library construction. Thereafter, end repair, A-tailing, and adaptor ligation were performed as described in the standard library construction protocol. These libraries were hybridized to a custom pool of oligonucleotides for target enrichment, and the probe panel was designed to cover cancer-specific genes in 593 tumor tissue samples and 418 plasma samples. Enriched libraries were amplified and sequenced using 2 × 150 bp paired-end reads on NovaSeq 6000 (Illumina, San Diego, CA, United States).

### Mutation analysis

Differences in exons were detected using a combination of depth of coverage and split-read analysis, supplemented with additional alignments generated using SLOPE. Frameshift, nonsense, missense, or splice site mutations of key genes predicted to be deleterious to protein function were validated using Sanger sequencing or droplet digital PCR, according to different allele mutation frequencies. The Yikon Genomics Panel has been proven to estimate TMB accurately. When 6–20 mutations per million bases in the tissue samples and 6–16 mutations per million bases in the body fluid samples were applied as the limits, the TMB level could be stratified into three tertiles: low, intermediate, and high.

The PD-L1 combined positive score was defined as the number of PD-L1-positive tumors and immune cells divided by the total number of tumor cells multiplied by 100. A value of one or greater was used to estimate PD-L1-positive tumors. MSI is evaluated based on the expression of mismatch repair genes and is linked to immunotherapy efficacy. Owing to the low benefit of immunotherapy in the MSI-L group, researchers developed the Yikon Genomics Panel combined MSI-L with the microsatellite stability (MSS) group and further defined MSI as two modes: MSI-H and MSS.

### Analysis and verification of pan-cancer samples in the database

The TMB and clinical data of pan-cancer samples from The Cancer Genome Atlas (TCGA) data portal were used as independent verification sets, and all cancer types with case projects greater than 100 were used for further analysis. Within each cancer type, the mutation status of key target genes and their effects on TMB and survival were elucidated. Furthermore, through integration with transcriptome data, Gene Set Enrichment Analysis (GSEA) identified significantly enriched pathways in the mutation group.

Thereafter, we mapped mutations and wild-type subsets of tumor patients to immune cell-related gene sets reported in previous studies and used single-sample GSEA to correlate the state of immune cell infiltration to further identify the changes in immune status caused by key target mutations. Furthermore, to investigate whether the genomic alterations in the key target genes were related to the response to immunotherapy, clinical cohorts with response annotations and matched mutational data from published studies were collected and consolidated. To verify our hypothesis, we divided these publicly accessible immunotherapy-treated patient cohorts into mutant and wild-type subgroups and analyzed the correlation between the mutation status and the clinical benefit of immunotherapy.

### Targeted therapy screening based on molecular docking

Small-molecule screening for the targeted intervention of key genes was achieved based on molecular docking analysis, using the Surflex-Dock Geom program interfaced with Sybyl. To qualify and filter the natural products in the ZINC database, SDF format files were downloaded, and further conformational enumerations and optimizations were performed. The X-ray crystal structure of the key proteins was extracted from the RCSB protein database, and the co-crystal ligands and structural water molecules were removed from the crystal structures before the docking simulation. Hydrogen atoms and Kollman total atomic charges were added and assigned. In our study, the ligand model was based on the binding site of the key target and receptor, thereby creating a binding pocket. If the original ligand existed, the structural similarity between the co-crystallized ligand and the target compound was also considered. Finally, effective intervention screening was realized based on binding strength and scoring.

### Statistical analysis

All statistical analyses were performed using GraphPad Prism 7.04 (San Diego, CA, United States) and SPSS 22.0 (IBM Corp., Armonk, NY, United States). Owing to the non-normal distribution of the TMB data, the differences between the two groups and between multiple groups were compared using the Wilcoxon Mann–Whitney test and Kruskal–Wallis test, respectively. Correlation analyses for TMB, mutation abundance and clinical characteristics were performed using Spearman’s linear regression. All p values were two-sided, and statistical significance was set at *P* ≤ 0.05.

## Results

### Analysis of the clinical and mutational characteristics of the patients who underwent next-generation sequencing

The demographic information of all 177 patients with tumors who underwent targeted NGS is summarized in Table 1. In the non-small cell lung cancer cohort, the majority of the patients who underwent NGS were male (35/56, 62.50%) with a median age of 61.77 years (range 40–85 years). The predominant sample types were tissue (26/56, 46.43%) and blood (27/56, 48.21%); however, there were a few samples from the hydrothorax and ascites (3/56, 5.36%). Only four samples could not be allocated a TMB score, and the TMB in the rest of the population ranged from 0–21.12 muts/Mb, with a median value of 5.66 muts/Mb. The number of patients classified as TMB-L, TMB-M, and TMB-H was 36, 14, and 2, respectively. The most common diagnosis in the pan-cancer cohort was colorectal cancer (18/121, 14.88%), followed by gastric cancer (17/121, 14.05%) and breast cancer (11/121, 9.09%) (Supplementary Table S1). They also showed a greater proportion of male patients and younger age groups, and the majority were TMB-L patients. The clinical outcomes suggest that most were stage III and IV patients as well as non-smokers; MSI status was also collected (Table 1).

**TABLE 1:** Clinical characteristics of cancer patients.

Characteristic	Non-small cell lung cancer cohort (*n* = 56)		Pan-cancer cohort (*n* = 121)	
	*n*	%	*n*	%
**Age, years**				
Mean	61.77		60.72	
Range	40–85		27–97	
≤65	39	69.64	72	59.50
>65	17	30.36	49	40.50
**Gender**				
Male	35	62.50	76	62.81
Female	21	37.50	45	37.19
**Sample type**				
Tissue	26	46.43	72	59.50
Blood	27	48.21	45	37.19
Hydrothorax and ascites	3	5.36	4	3.31
**TMB**				
Mean	5.66		8.01	
Range	0–21.12		0–103.7	
TMB-L	36	64.29	73	60.33
TMB-M	14	25.00	29	23.97
TMB-H	2	3.57	13	10.74
NA	4	7.14	6	4.96
**MSI**				
MSS	38	67.86	85	70.25
MSI-H	0	0	4	3.31
NA	18	32.14	32	26.44
**Smoking**				
True	10	17.86	27	22.31
False	19	33.93	53	43.80
NA	27	48.21	41	33.89

TMB, tumor mutation burden; MSI, microsatellite instability; MSS, microsatellite stability.

**FIGURE 2:**
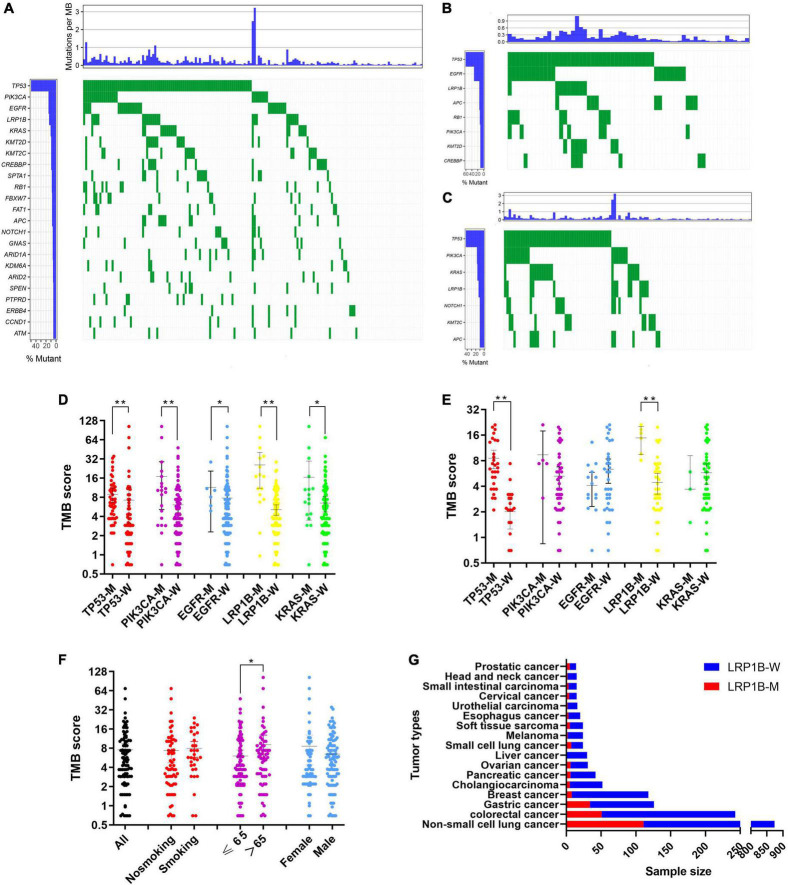
Screening and characterization of key genetic mutations. **(A)** A waterfall map describing genetic mutations appearing with a greater than 5% frequency in total 177 patients; **(B,C)** waterfall maps describing genetic mutations appearing with a greater than 10% frequency in non-small cell lung cancer cohort and pan-cancer cohort respectively; **(D,E)** Comparison of TMB in mutant and wild-type subgroups for *TP53*, *EGFR*, *PIK3CA*, *LRP1B*, and *KRAS* in non-small cell lung cancer cohort and pan-cancer cohort respectively; **(F)** Correlation between demographic or clinicopathological features and TMB. Error bars represent the mean with a 95% CI; **(G)** Comparison of *LRP1B* mutation rates in an additional 1683 pan-cancer clinical samples. LRP1B-M: *LRP1B* mutation, LRP1B-W: *LRP1B* wild-type. *p < 0.05, **p < 0.01.

The mutation profiles for all 177 patients are summarized in Figure 2A, while the mutation details of each of the two cohorts are shown in Figures 2B,C. *TP53* showed the highest mutation rate, followed by *PIK3CA*, *EGFR*, *LRP1B*, and *KRAS*. It has a commonality with the mutation details of each of the two queues. Furthermore, only 19.77% (35/177) of patients had at least one clinically relevant genomic alteration. We evaluated the mutational characteristics of *TP53*, *PIK3CA*, *EGFR*, *LRP1B*, and *KRAS* using the Catalogue of Somatic Mutations in Cancer (COSMIC^1^) database. COSMIC is the world’s largest and most comprehensive resource for exploring the impact of somatic mutations on human cancers. A description of the mutation type and details of its frequencies for each of the five genes, based on a large sample set in this database, is provided in Supplementary Figure S1.

### Clinical treatment guided by next-generation sequencing

A total of 39 patients received NMPA-approved immunization or targeted medication evaluations. In 24 of these patients, this resulted in a change to their clinical management, and of these, only four (16.67%) received standard targeted therapy guided by gene mutation. The remaining patients were further subdivided as follows: 17 (70.83%) who received immunotherapy based on their TMB status (medium or high) and 3 (12.5%) who received both therapies in subsequent rounds of administration. Of the 24 patients, half were lung cancer, and more than half of them (58.33%) had previously received less than three treatments of chemotherapy, with the highest number calculated at 25. The average number of chemotherapy treatments received prior to enrolment in this study was 4.875. The patient information and clinical outcomes of those who underwent clinical management changes based on their NGS profiles are described in Supplementary Table S2. These clinical management changes refer to the discovery of targeted therapy or immunotherapy recommended by the guidelines based on the NGS results, which were then administered to these patients. Sixteen patients experienced a documented clinical benefit following the revision of their treatment plan. In addition, when we evaluated the disclosed hospitalization costs of 15 of these patients, the average number of days of hospitalization between the last chemotherapy and first treatment following NGS-guided changes was 15.4 (coefficient of variation: 1.70) and 16.5 (coefficient of variation: 1.25) days, respectively. There were no increases in the daily drug costs before and after these changes (¥1142.37 vs. ¥1312.15, *p* > 0.05).

### Comparing tumor mutational burden values between subgroups

We evaluated the influence of different subgroups on TMB. Because tumors harboring different gene mutations may have distinct biological behaviors, we divided our data into subgroups based on their primary gene mutations, *TP53*, *PIK3CA*, *EGFR*, *LRP1B*, and *KRAS*. These results demonstrated that all five genes showed increased TMB levels in the mutation group of the pan-cancer cohort (Figure 2D), while only *TP53* and *LRP1B* showed this increase in the non-small cell lung cancer cohort (Figure 2E). Participants with mutations in *LRP1B* exhibited medium to high TMB values. The patients older than 65 years showed increased TMB expression rates within their subgroups (Figure 2F).

Clinical examination indices, including routine blood tests, liver and kidney function tests, and tumor biomarker evaluations, were collected, and their possible correlation with TMB was evaluated (Table 2). Using Spearman correlation analysis, we found that ferritin (379.59 ± 462.09, *p* = 0.006), neuron-specific enolase (NSE; 20.59 ± 10.77, *p* = 0.006), hematocrit (34.79 ± 6.95, *p* = 0.032), mean corpuscular hemoglobin concentration (329.41 ± 11.35, *p* = 0.015), albumin levels (40.18 ± 4.90, *p* = 0.003), and total bile acid (8.94 ± 30.38, *p* = 0.019) were strongly associated with changes in TMB value. The curve-fitting diagram constructed based on locally weighted scatterplot smoothing (Lowess) is shown in Supplementary Figure S2.

**TABLE 2:** Potential correlations between laboratory test results and TMB.

No.	Term	Values (mean ± SD)	Correlation with TMB (Spearman test)
**Complete blood Count**			
1	WBC	7.17 ± 5.65	*p* = 0.824
2	Neu%	68.58 ± 12.64	*p* = 0.320
3	Neu#	5.29 ± 5.25	*p* = 0.817
4	LY%	22.50 ± 11.06	*p* = 0.178
5	LY#	1.29 ± 0.59	*p* = 0.137
6	Mon%	6.76 ± 3.77	*p* = 0.363
7	Mon#	0.42 ± 0.22	*p* = 0.340
8	Eos%	1.82 ± 1.65	*p* = 0.076
9	Eos#	0.14 ± 0.33	*p* = 0.060
10	Bas%	0.32 ± 0.24	*p* = 0.689
11	Bas#	0.02 ± 0.02	*p* = 0.959
12	RBC	3.83 ± 0.82	*p* = 0.172
13	HGB	114.80 ± 23.68	*p* = 0.167
14	HCT	34.79 ± 6.95	*p = 0.032
15	MCV	91.60 ± 6.90	*p* = 0.408
16	MCH	30.18 ± 2.71	*p* = 0.883
17	MCHC	329.41 ± 11.35	*p = 0.015
18	RDW-SD	47.37 ± 9.87	*p* = 0.893
19	RDW-CV	14.05 ± 2.03	*p* = 0.743
20	PLT	238.61 ± 99.4	*p* = 0.580
21	PCT	0.20 ± 0.07	*p* = 0.352
22	MPV	8.86 ± 1.21	*p* = 0.772
23	PDW	15.97 ± 0.54	*p* = 0.506
24	P-LCR	18.32 ± 7.65	*p* = 0.786
**Basic metabolic tests**			
25	K	4.10 ± 0.36	*p* = 0.163
26	Na	136.87 ± 21.32	*p* = 0.651
27	CI	100.92 ± 5.11	*p* = 0.271
28	CO_2_	24.90 ± 2.81	*p* = 0.414
29	CA	2.34 ± 0.27	*p* = 0.318
**Liver function tests**			
30	ALT	34.80 ± 45.33	*p* = 0.181
31	AST	47.86 ± 123.12	*p* = 0.333
32	AST/ALT	1.42 ± 0.87	*p* = 0.882
33	ALP	130.20 ± 175.07	*p* = 0.059
34	GGT	121.41 ± 384.65	*p* = 0.128
35	TP	66.97 ± 7.59	*p* = 0.205
36	ALB	40.18 ± 4.90	**p = 0.003
37	GLO	26.80 ± 4.71	*p* = 0.876
38	A/G	1.54 ± 0.33	*p* = 0.178
39	TBIL	13.11 ± 11.72	*p* = 0.237
40	DBIL	3.83 ± 8.56	*p* = 0.417
41	IBIL	9.28 ± 4.83	*p* = 0.073
42	TBA	4.46 ± 6.30	*p = 0.030
**Renal function tests**			
43	UA	299.45 ± 98.14	*p* = 0.177
44	UREA	5.16 ± 1.87	*p* = 0.946
**Tumor markers**			
45	CR	65.24 ± 22.56	*p* = 0.861
46	GLU	5.87 ± 1.66	*p* = 0.101
47	CEA	97.51 ± 458.65	*p* = 0.423
48	CA125	66.85 ± 151.64	*p* = 0.266
49	CYF211	10.05 ± 12.82	*p* = 0.681
50	NSE	20.59 ± 10.77	**p = 0.006
51	CA199	294.87 ± 777.89	*p* = 0.127
52	CA724	22.01 ± 57.27	*p* = 0.115
53	Ferritin	379.59 ± 462.09	**p = 0.006
54	Ki67	62.22 ± 22.92	*p* = 0.473
**Blood coagulation tests**			
55	PT-SEC	12.63 ± 1.38	*p* = 0.811
56	INR	1.06 ± 0.11	*p* = 0.959
57	PT-%	90.58 ± 12.22	*p* = 0.959
58	APTT	29.15 ± 4.59	*p* = 0.639
59	TT	15.93 ± 1.74	*p* = 0.191
60	Fib	3.66 ± 1.30	*p* = 0.464
61	DD	1.83 ± 4.12	*p* = 0.216
**Myocardial zymogram**			
62	LDH	218.17 ± 95.71	*p* = 0.072
63	CK	44.67 ± 28.05	*p* = 0.257
64	CK-MB	32.33 ± 46.01	*p* = 0.957
65	HBDH	171.00 ± 71.68	*p* = 0.072
66	CHO	5.33 ± 1.50	*p* = 0.068
67	TG	1.17 ± 0.46	*p* = 0.577
68	HDL	1.41 ± 0.53	*p* = 0.195
69	LDL	2.66 ± 0.97	*p* = 0.217

*p < 0.05, **p < 0.01. WBC, white blood cell; Neu, neutrophil; LY, lymphocyte; Mon, monocyte; Eos, eosinophil; Bas, basophil; RBC, red blood cell; HGB, hemoglobin; HCT, hematocrit; MCV, mean corpuscular volume; MCH, mean corpuscular hemoglobin; MCHC, mean corpuscular hemoglobin concentration; RDW, red blood cell distribution width; PLT, platelet; PCT, plateletcrit; MPV, mean platelet volume; PDW, platelet distribution width; P-LCR, platelet-large cell ratio; ALT, alanine aminotransferase; AST, aspartate aminotransferase; ALP, alkaline phosphatase; GGT, gamma glutamyl transferase; TP, total protein; ALB, albumin; GLO, globulin; TBIL, total bilirubin; DBIL, direct bilirubin; IBIL, indirect bilirubin; TBA, total bile acid; UA, uric acid; CR, creatinine; GLU, glucose; CEA, carcinoembryonic antigen; NSE, neuron-specific enolase; PT, prothrombin time; INR, international normalised ratio; APTT, activated partial thromboplastin time; TT, thrombin time; FiB, fibrinogen; DD, D-dimer; LDH, lactate dehydrogenase; CK, creatine kinase; HBDH, α-hydroxybutyrate dehydrogenase; CHO, cholesterol; TG, triglyceride; HDL, high-density lipoprotein; LDL, low-density lipoprotein.

### Analysis of key mutation targets based on verification sets

To further explore the characteristics of the mutations, 25 solid tumor datasets with case studies greater than 100 were downloaded, and 9475 mutation samples were used for further analysis. The mutation states of the five key targets and the mutation characteristics of each cancer type were captured (Supplementary Figure S3 and Supplementary Table S3). The results showed that among the five key genes, *TP53* had the highest mutation frequency, reaching 35.04% (3320/9475); followed by *PIK3CA* (12.78%, 1211/9475), *LRP1B* (11.01%, 1043/9475), *KRAS* (6.51%, 617/9475), and *EGFR* (3.40%, 322/9475). This was consistent with the results of pan-cancer data obtained from large samples in the COSMIC database (Supplementary Figure S1). In addition, the mutation states of the targets in different cancer species led to different trends in TMB expression (Supplementary Figure S4). Mutations in *TP53*, *PIK3CA*, *EGFR*, and *KRAS* were often accompanied by a high TMB status in the tumor, while the special cases were the *TP53* mutant group in uterine corpus endometrial carcinoma and the *EGFR* mutant group in lung adenocarcinoma (LUAD) and lung squamous cell carcinoma (LUSC). In addition, the survival state was analyzed based on the clinical data of each cancer type (Supplementary Table S4). Further survival analysis was conducted by integrating the gene expression profiles and clinical information from the Genotype-Tissue Expression Portal, and the risk ratios were calculated (Supplementary Figure S5).

### Exploration of typical mutants in the *LRP1B* gene

**FIGURE 3:**
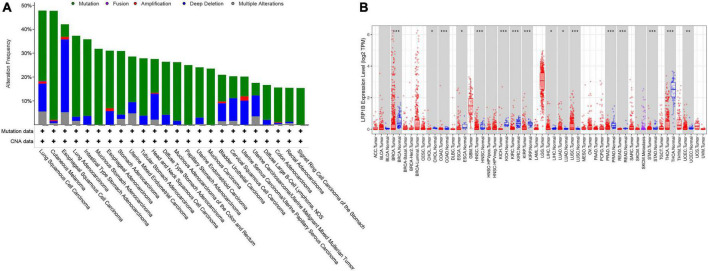
Expression and mutation characteristics of *LRP1B* in different cancer species. **(A)** Display of 24 cancer species with *LRP1B* mutation frequency greater than 15%; **(B)** Differential expression of *LRP1B* in 32 cancer subtypes and/or corresponding normal tissues (gray columns) where normal data are available. *p < 0.05, **p < 0.01, and ***p < 0.001.

A significant correlation between the *LRP1B* mutation and TMB was observed, with the TMB values for the majority of the patients (20/22, 90.91%) and the *LRP1B* mutation falling into the medium or high TMB categories (Figures 2D,E). Given the relative novelty of the mutations in *LRP1B* compared to those in the other four genes, we expanded the analysis of this gene to include 1683 more clinical samples. These results revealed that the cancer subtype with the highest *LRP1B* mutation rate was prostate cancer (5/14, 35.7%), followed by small-cell lung cancer (7/24, 29.2%), gastric cancer (34/126, 27.0%), and cervical cancer (4/15, 26.7%); the average mutation rate was observed to be 15.2% (255/1683) (Figure 2G and Supplementary Table S5). This differed from the outcome of large pan-cancer samples in the cBioPortal database^2^, which may be attributed to the false-positive expression due to the small sample size of our “real-world” tumor data (Figure 3A).

Further database-based verification and analysis of *LRP1B* were performed. The nucleotide variation and clinical data from 25 solid tumors were obtained from TCGA. The results suggested that *LRP1B* displayed a high mutation frequency in skin cutaneous melanoma (37.55%), LUAD (30.08%), LUSC (29.24%), and stomach adenocarcinoma (23.67%); this was consistent with the results obtained from the cBioPortal database. Survival analysis was based on both the transcriptome and genome. In addition, transcriptome expression data were downloaded and transformed into more standardized TPM data to facilitate between-sample comparisons (Figure 3B).

Kyoto Encyclopedia of Genes and Genomes (KEGG)-derived gene sets, collected from MSigDB C2, were used in GSEA. We aligned TCGA data and focused on a single gene, *LRP1B*, for the phenotype. Pathways such as “MISMATCH REPAIR,” “BASAL TRANSCRIPTION FACTORS,” and “DNA REPLICATION” were significantly enriched (Supplementary Figure S6 and Supplementary Table S6). In addition, mutation of *LRP1B* led to a variety of metabolic changes. Notably, the synthesis and metabolic pathways of folic acid were significantly enriched, which may explain the relationship between TMB and mean corpuscular hemoglobin concentration to some extent.

### Benefit analysis of immunotherapy for *LRP1B* mutant subsets

**FIGURE 4:**
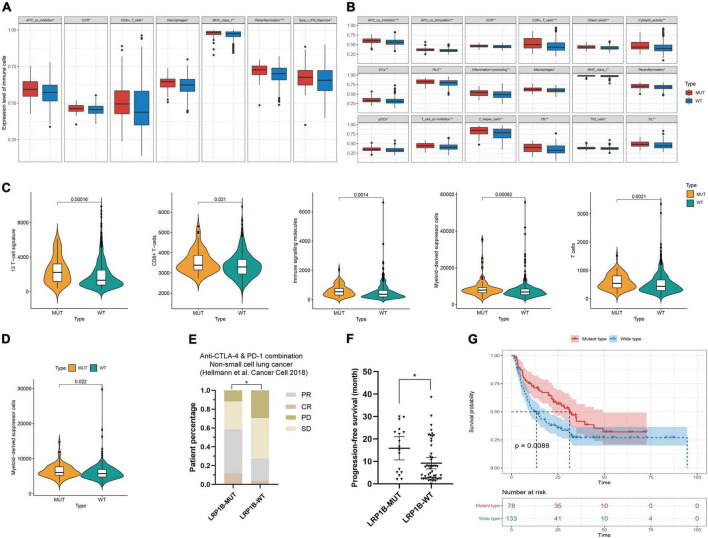
Characteristics of immune infiltration and benefit analysis of immunotherapy for *LRP1B* mutant subsets. **(A–D)** Immunocyte association analysis in lung adenocarcinoma and lung squamous cell carcinoma based on ssGSEA and reported immune cell-related gene sets, respectively; **(E,F)** The patients harboring *LRP1B* mutation had a better objective response rate and a longer progress free survival in the non-small cell lung cancer cohort of Hellmann; **(G)** Melanoma population with *LRP1B* mutant showed better overall survival after immunotherapy. *p < 0.05, **p < 0.01, ***p < 0.001.

The immunocyte association analysis of single-sample GSEA in non-small cell lung cancer showed that the *LRP1B* mutation led to a high level of immune cell infiltration. The mutant subsets exhibited extensive enrichment of immune cells, such as T cells, MHC class I, chemokine receptors, and macrophages (Figures 4A,B); the same outcome was obtained based on the immune cell-related gene sets reported previously (Figures 4C,D). Furthermore, the high level of immune infiltration in the *LRP1B* mutant population seems to be more prominent in LUSC than in LUAD.

The outcomes of mutations showed that the *LRP1B* mutant group appeared to be associated with immunotherapy benefits. To investigate this association, clinical cohorts with response annotations and matched mutational data obtained from previous studies were collected and consolidated. We divided these publicly accessible, immunotherapy-treated patient cohorts into the LRP1B-MUT and LRP1B-WT subgroups and analyzed the correlation between *LRP1B* mutation status and clinical immunotherapy benefit. In the non-small cell lung cancer cohort of Hellmann et al. (13), the patients harboring *LRP1B* mutation demonstrated an enhance objective response rate (ORR) and progression-free survival (PFS), the results were statistically significant (Figures 4E,F). A similar outcome was observed in the melanoma cohort, wherein the *LRP1B* mutant population showed better overall survival (OS) after immunotherapy (Figure 4G). The potential clinical implications were stratified by the specific *LRP1B* mutation state.

### *APC* mutation characteristics analysis and screening of targeted intervention molecules

In this study, we identified the mutation of the key target *APC* in both the non-small cell lung cancer and pan-cancer cohort; however, the analysis showed that it was not associated with TMB and did not provide any immunotherapy benefit. As a typical tumor suppressor gene, *APC* can negatively regulate the canonical WNT signaling pathway and participate in the regulation of cell-cell adhesion and cell migration by recognizing and activating Asef (14). In further GSEA-based pathway analysis of non-small cell lung cancer and colorectal cancer, pathways closely related to tumorigenesis and development were identified, such as “Regulation of intrinsic apoptotic signaling pathway in response to DNA damage” and “Response to misfolded protein” (Supplementary Table S7). Therefore, we considered the potential benefits of small-molecule targeted therapy rather than those of immunotherapy.

**FIGURE 5:**
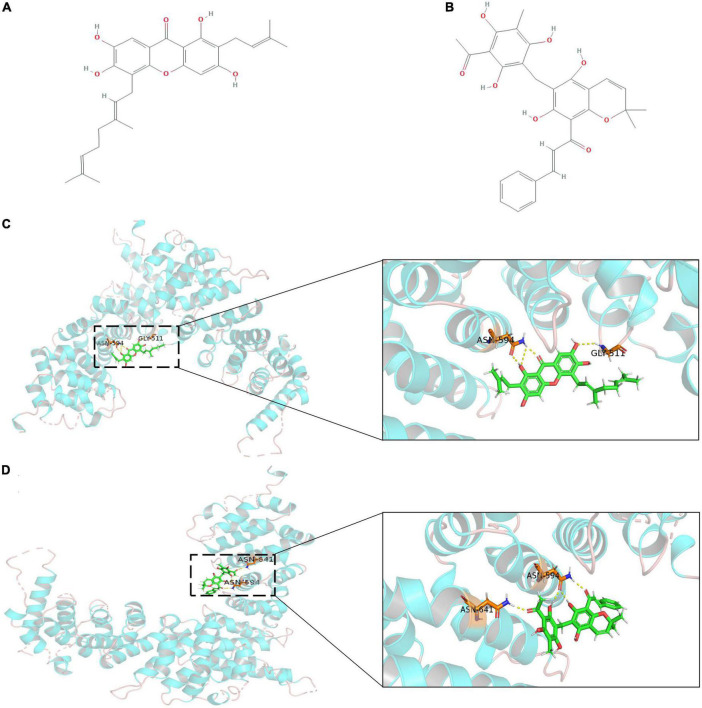
The outcome of molecular docking between APC and key compounds. **(A,B)** Chemical structure depiction of Cochinchinone B and Rottlerin; **(C,D)** Whole and partial display of molecular docking. The X-ray crystal structure of APC protein was used as receptor protein and molecules are present as ball and stick models. The dotted yellow lines in these pictures represent H-bonds, while the docking nucleotide sites were also displayed.

Using the ZINC database, 224,205 natural products and 3,725 small molecular structures with clear identification and *in vitro* activity were downloaded and saved in mol2 format for further molecular docking analysis. The X-ray crystal structure of APC (PDB ID: 3 NMW) was used as the receptor protein. Furthermore, we generated active pockets to achieve the effective docking of small molecules. Docking results showed that the total scores of 57 small molecules were higher than 9, of which 15 components scored higher than 10 (Supplementary Table S8). We further analyzed previous publications regarding these 15 components to determine whether they were clearly associated with tumors. Finally, the key components, cochinchinone B and rottlerin, were identified, and their spatial structure and hydrogen bonding sites were revealed based on PyMOL visualization (Figure 5).

## Discussion

In recent years, NGS has played a pivotal role in the process of solid tumor treatment becoming individualized. Personalized therapeutics based on various molecular markers are becoming increasingly popular in both research and clinical settings because of their high efficacy and reduced side effects (15). Although the number of cancers sequenced has increased exponentially, fewer patients are amenable to NGS-directed targeted therapy or immunotherapy. The understanding of the biological significance of genetic alterations still needs to be improved. In this study, we collected information from 177 patients who underwent NGS. We identified the mutation characteristics of the two cohorts and analyzed the association between TMB, genetic alteration, and clinical information. Importantly, we identified and analyzed the key regulatory roles of several indicators, particularly *LRP1B* and *APC*. *LRP1B* mutations were frequently accompanied by higher TMB scores in both non-small cell lung cancer and pan-cancer cohorts and led to better immune benefits, thereby suggesting that *LRP1B* mutation stratification may play a guiding role in immunotherapy. Moreover, we identified *APC* mutations. Owing to the low immune correlation, we focused on potential targeted interventions. We determined that cochinchinone B and rottlerin may be effective interventions by screening a large number of small molecular compounds.

Tumor mutational burden has been effectively defined by the rapid development of NGS and shown to be a reasonably effective marker for patients who may benefit from immunotherapy as part of their course of treatment. Many studies have demonstrated a strong correlation between TMB and clinical management, especially immunotherapy (16). In the present study, we provide new insights into the interaction between TMB, different patient characteristics, and clinical indicators. First, patients older than 65 years demonstrated increased TMB expression rates; a similar trend was also observed in smokers. This result is with the research of Lin et al. (17). Second, our results showed a correlation between TMB and several clinical markers, including ferritin and NSE. In addition, we observed that TMB correlates with several biochemical markers, including hematocrit and albumin; however, because of the limitations in sample size, these results need to be interpreted with caution.

The five most frequently mutated genes, *TP53*, *PIK3CA*, *EGFR*, *LRP1B*, and *KRAS*, were identified; we were able to show that there were differences in the TMB values between the mutant and wild-type populations. With the exception of *EGFR*, all genes demonstrated increased TMB levels in the mutant group of the non-small cell lung cancer cohort. This is consistent with the molecular epidemiology data that suggest that non-small cell lung cancer individuals with *EGFR* mutations often present with an “immune-desert” phenotype, where no inflammation is observed within the tumor microenvironment along with low overall TMB, immunological tolerance, and weak immunogenicity (18). We focused on *LRP1B* because its mutation rate in this population was approximately 16% (altered/profiled = 255/1683, Supplementary Table S5). In our datasets, the TMB rates in samples with *LRP1B* mutations tended to fall into the medium or high categories, and further analysis of 25 independent cancer types verified this outcome, thereby suggesting that *LRP1B* mutations may be a good biomarker for immune intervention. A series of verifications based on a clinical immunotherapy cohort supported and validated the hypothesis that after receiving immunotherapy, patients in the *LRP1B* mutation group showed higher ORR, longer OS and PFS. This significant immune benefit was observed in patients with both non-small cell lung cancer and melanoma.

We discovered an effective *APC* mutation in two cancer cohorts. As a classic tumor suppressor gene, inactivated mutations in *APC* are thought to trigger the “adenomato-carcinoma sequence.” Adenoma is the most common precancerous lesion in almost all sporadic colorectal cancer; the “adenomato-carcinoma sequence” plays an important role in the development of colorectal cancer. *APC* participates in a cytoplasmic complex that promotes the phosphorylation and ubiquitination of the transcription factor β-catenin and negatively regulates the canonical WNT signaling pathway. In addition, *APC* mutations usually occur as truncation C-terminal mutations in its mutation cluster region, resulting in defective regulation of β-catenin phosphorylation and ubiquitination, but potentially increasing activation of Asef by the ARM domain (19). Therefore, it is necessary to explore effective targeted interventions for mutant *APC*. For our study, we constructed an active pocket to recognize the binding efficiency of small molecules in the armadillo repeat domain of APC protein, the potential intervention effect of cochinchinone B and rottlerin were determined *via* independent docking with 3725 small molecules. The cochinchinone B is a polyphenol found in *Cratoxylum cochinchinense*, and rottlerin is a natural polyphenolic compound found in *Mallotus philippensis* that can be used as an inhibitor of various proteins. cochinchinone B and rottlerin exert antitumor biological activities in many tumor cells and may provide a potential intervention in cancer, especially gastrointestinal tumors.

In addition to the continued exploration and analysis at the molecular level, the practical clinical significance of NGS needs further interpretation. In our study, NGS identified at least one potential clinically actionable genomic alteration, where targeted drugs are marketed in China and approved by the National Medical Products Administration. However, this alteration was found in only 35 patients (19.77%), which is far below the level of other more developed countries (20). Of the 24 patients who received NGS-guided treatment, only 7 received targeted therapy, whereas the majority of patients received NGS-guided immunotherapy. However, 16 of these patients did derive some clinical benefits from NGS-guided treatment changes. These data should be improved because there is a situation in which the samples that some patients choose to undergo NGS after a period of treatment or progression of the disease are those from the beginning of the disease several months or years ago. Identifying the potential molecular mechanisms underlying the pathology and progression currently requires synchronized analysis of primary tumors to avoid the noise associated with the evolution of its genome over time.

Of equal importance are the clinical costs. Although our results show that neither the total hospitalization cost (¥15893.91 vs. ¥19976.25, *p* > 0.05) nor the average daily cost (¥1142.37 vs. ¥1312.15, *p* > 0.05) was different before and after the NGS-guided treatment, it should be noted that this was the total cost before reimbursement of medical insurance. The cost of chemotherapy before sequencing can be fully reimbursed by medical insurance on a pro rata basis; however, the cost of targeted drugs and immune preparations is borne by the patients, which may result in changes to the cost outcomes described here. In addition, the cost of NGS is also borne by the patients and remains expensive when using some of the comprehensive cancer panels. The average cost is approximately ¥15000, making this approach less feasible in economically restricted environments. The first study to report the cost of anticancer drugs for matched and unmatched treatments using NGS in cancer patients found that patients who were treated with NGS-guided treatment had a higher total treatment cost ($68,729 vs. $30,664; *p* = 0.003), but the drug costs were largely attributed to longer treatment times rather than higher monthly drug costs (21). However, the purely economic argument should be tempered by the benefits of NGS-guided treatment that may outweigh the increased costs; these include better clinical efficacy and more stable hospitalization cycles (coefficient of variation for hospitalization was 1.70 pre-treatment and 1.25 post-treatment).

Next-generation sequencing has successfully guided the choice of clinically targeted therapies and immunotherapies, thereby expanding the application of these drugs beyond the current limitations associated with cancer variants and pathological subtypes and providing a new understanding of cancer management. However, clinical outcomes guided by NGS alone are still unsatisfactory (22). As previously mentioned, the interval between the acquisition of samples and the receipt of NGS data may be long, making the data irrelevant for cancers with fast progression. In principle, additional NGS analysis and re-sampling should be routinely applied to identify novel variations associated with tumor progression in patients (23). Re-sampling and sequencing can more accurately guide treatment; however, the actual clinical situation is often not conducive to this approach, with the high cost of NGS and the traumatic re-acquisition of pathological tissues. Non-invasive liquid biopsies to detect circulating tumor DNA (ctDNA), can overcome the limitations of tissue sequencing and capture the biological heterogeneity of cancer as well as the dynamic adaptation to anticancer therapies; thus, making this approach increasingly attractive to clinicians (24). However, some practical challenges are associated with ctDNA assays, including the concentration and stability of ctDNA, purification of these nucleic acids, and degradation of these biomarkers; these challenges limit their efficacy in the clinic (25, 26). Additionally, the process of sample collection and isolation is an important factor. Improper collection, transportation, and storage can increase degradation and reduce the resolution of these assays (27). NGS evaluation in larger panels often takes up to a week, and prolonged NGS processing may force patients to start ineffective and/or toxic treatments before receiving their results (28). Therefore, it is critical to identify novel biomarkers that can be assayed quickly to differentiate between various conditions (29). Screening high-sensitivity mutations, reducing panel sets, and constructing a comprehensive biomarker model beyond the univariate versions currently being used may help bring these breakthroughs to the clinic.

Despite generating some positive initial results, our study has several limitations. Some of the conclusions may be limited by the sample size and follow-up time, which were insufficient to evaluate long-term survival benefits and any additional therapeutic changes implicated by additional NGS data. However, we discovered the clinical benefits of NGS-guided treatment, and our results were similar to those reported by Singh et al. (20) and Marquart et al. (30). Most of the patients in these cohorts were later-line, meaning that the CGP of these patients in the earlier stages was underrepresented. In addition, our data were obtained from a single institution, which increased selection bias but ensured the uniformity of the data measurement standards. Notably, improvements in therapeutic management for cancer patients often lag behind the breakthroughs in driver gene treatment. Thus, we aim to analyze a larger sample size with a longer follow-up period, which should provide more detailed genetic profiles and clinical information for NGS-guided treatment in ‘real-world’ patients. Further in-depth research on key biomarkers will eventually reduce the differences in treatment among people with specific molecular characteristics.

## Conclusion

Our study provided the characteristics of specific gene mutations and demonstrated the benefits of NGS for clinical strategy development. Immunotherapy accounted for the majority of management changes in this cohort, and TMB was shown to play an important role in the genetic mapping of the clinical response. The effect of *LRP1B* mutation stratification on immune benefit and potential targeted intervention of *APC* were identified. In conclusion, associating molecular profiles to clinical benefits in ‘real-world’ patients is the ultimate goal of personalized treatment.

## Data availability statement

The datasets presented in this study can be found in online repositories. The names of the repository/repositories and accession number(s) can be found below: https://www.ncbi.nlm.nih.gov/, PRJNA721133.

## Ethics statement

The studies involving human participants were reviewed and approved by The protocol was approved by the Ethics Committee at the Weifang Traditional Chinese Hospital (No. 2021-WFRS-006). The patients/participants provided their written informed consent to participate in this study.

## Author contributions

CL, YY, and JZ had the idea for and designed this study. GW, JL, and YL had full access to all the data in this study. RL, LL, and XY analyzed and interpreted the experimental data. HL and CG analyzed the samples, designed the study, and interpreted the data. CG and JZ took the responsibility for double checking of the data analysis. CL, YL, and JZ performed the statistical analysis and designed the figures and tables. CL, YY, and GW wrote the manuscript. All authors read and revised the manuscript, contributed to discussion, and approved the final version of this manuscript.

## Footnotes

1
https://cancer.sanger.ac.uk/cosmic

2
http://www.cbioportal.org

## Supplementary material

Supplementary Figure S1 An overview of the types of mutation and breakdown of the substitution mutations at the five most common mutation sites in this study. **(A–C)** *TP53*; **(D–F)** *EGFR*; **(G–I)** *PIK3CA*; **(J–L)** *LRP1B*; **(M–O)** *KRAS*.

Supplementary Figure S2 Curve fitting correlation between TMB and clinical indicators. Unadjusted data is fitted according to locally weighted scatterplot smoothing, showing the trend of association between data. Especially with the increase of NSE and ferritin, the level of TMB increased significantly. MCHC, mean corpuscular hemoglobin concentration; ALB, albumin; TBA, total bile acid; NSE, neuron-specific enolase.

Supplementary Figure S3 Analysis of tumor mutation characteristics based on TCGA Database. **(A)** Further analysis of the ten key sites with the highest mutation rate in each solid tumors (*n* = 25); **(B)** Mutation characteristics analysis of 9475 samples, the top 15 genes with the highest mutation frequency were displayed in waterfall map.

Supplementary Figure S4 The correlation between key targets (the top 10 sites with the highest mutation rate of 25 solid tumors and 5 key mutation sites identified in clinical samples) and TMB level in various tumors.

Supplementary Figure S5 Survival risk analysis of 5 key targets, risk ratios were calculated based on expression and clinical information. The primary sarcomas in retroperitoneum, peritoneum, connective, subcutaneous and other soft tissues are summarized and collectively referred to as SARC.

Supplementary Figure S6 KEGG derived gene sets were used to implement GSEA, which focused on single gene-LRP1B for the phenotype.

## References

[1] Bray F Ferlay J Soerjomataram I Siegel RL Torre LA Jemal A. Global cancer statistics 2018: GLOBOCAN estimates of incidence and mortality worldwide for 36 cancers in 185 countries. *CA Cancer J Clin.* (2018) 68:394–424. 10.3322/caac.21492 30207593

[2] Brown NA Elenitoba-Johnson KSJ. Enabling precision oncology through precision diagnostics. *Annu Rev Pathol.* (2020) 15:97–121. 10.1146/annurev-pathmechdis-012418-012735 31977297

[3] Weinstein JN Collisson EA Mills GB Shaw KR Ozenberger BA Ellrott K The cancer genome atlas pan-cancer analysis project. *Nat Genet.* (2013) 45:1113–20. 10.1038/ng.2764 24071849 PMC3919969

[4] Berger MF Mardis ER. The emerging clinical relevance of genomics in cancer medicine. *Nat Rev Clin Oncol.* (2018) 15:353–65. 10.1038/s41571-018-0002-6 29599476 PMC6658089

[5] Frampton GM Fichtenholtz A Otto GA Wang K Downing SR He J Development and validation of a clinical cancer genomic profiling test based on massively parallel DNA sequencing. *Nat Biotechnol.* (2013) 31:1023–31. 10.1038/nbt.2696 24142049 PMC5710001

[6] Goldberg KB Blumenthal GM Pazdur R. The first year of the food and drug administration oncology center of excellence: landmark approvals in a dynamic regulatory environment. *Cancer J.* (2018) 24:131–5. 10.1097/ppo.0000000000000316 29794538

[7] Skoulidis F Heymach JV. Co-occurring genomic alterations in non-small-cell lung cancer biology and therapy. *Nat Rev Cancer.* (2019) 19:495–509. 10.1038/s41568-019-0179-8 31406302 PMC7043073

[8] Slamon DJ Leyland-Jones B Shak S Fuchs H Paton V Bajamonde A Use of chemotherapy plus a monoclonal antibody against HER2 for metastatic breast cancer that overexpresses HER2. *N Engl J Med.* (2001) 344:783–92. 10.1056/nejm200103153441101 11248153

[9] Vyas S Chang P. New PARP targets for cancer therapy. *Nat Rev Cancer.* (2014) 14:502–9. 10.1038/nrc3748 24898058 PMC4480224

[10] Sicklick JK Kato S Okamura R Schwaederle M Hahn ME Williams CB Molecular profiling of cancer patients enables personalized combination therapy: the I-PREDICT study. *Nat Med.* (2019) 25:744–50. 10.1038/s41591-019-0407-5 31011206 PMC6553618

[11] Hulsen T Jamuar SS Moody AR Karnes JH Varga O Hedensted S From big data to precision medicine. *Front Med.* (2019) 6:34. 10.3389/fmed.2019.00034 30881956 PMC6405506

[12] Singal G Miller PG Agarwala V Li G Kaushik G Backenroth D Association of patient characteristics and tumor genomics with clinical outcomes among patients with non-small cell lung cancer using a clinicogenomic database. *JAMA.* (2019) 321:1391–9. 10.1001/jama.2019.3241 30964529 PMC6459115

[13] Hellmann MD Nathanson T Rizvi H Creelan BC Sanchez-Vega F Ahuja A Genomic features of response to combination immunotherapy in patients with advanced non-small-cell lung cancer. *Cancer Cell.* (2018) 33:843–852.e4. 10.1016/j.ccell.2018.03.018 29657128 PMC5953836

[14] Kawasaki Y Sato R Akiyama T. Mutated APC and Asef are involved in the migration of colorectal tumour cells. *Nat Cell Biol.* (2003) 5:211–5. 10.1038/ncb937 12598901

[15] Mosele F Remon J Mateo J Westphalen CB Barlesi F Lolkema MP Recommendations for the use of next-generation sequencing (NGS) for patients with metastatic cancers: a report from the ESMO Precision Medicine Working Group. *Ann Oncol.* (2020) 31:1491–505. 10.1016/j.annonc.2020.07.014 32853681

[16] McNamara MG Jacobs T Lamarca A Hubner RA Valle JW Amir E. Impact of high tumor mutational burden in solid tumors and challenges for biomarker application. *Cancer Treat Rev.* (2020) 89:102084. 10.1016/j.ctrv.2020.102084 32738738

[17] Lin C Shi X Zhao J He Q Fan Y Xu W Tumor mutation burden correlates with efficacy of chemotherapy/targeted therapy in advanced non-small cell lung cancer. *Front Oncol.* (2020) 10:480. 10.3389/fonc.2020.00480 32411590 PMC7201001

[18] Dong ZY Zhang JT Liu SY Su J Zhang C Xie Z EGFR mutation correlates with uninflamed phenotype and weak immunogenicity, causing impaired response to PD-1 blockade in non-small cell lung cancer. *Oncoimmunology.* (2017) 6:e1356145. 10.1080/2162402x.2017.1356145 29147605 PMC5674946

[19] Zhang Z Chen L Gao L Lin K Zhu L Lu Y Structural basis for the recognition of Asef by adenomatous polyposis coli. *Cell Res.* (2012) 22:372–86. 10.1038/cr.2011.119 21788986 PMC3271586

[20] Singh AP Shum E Rajdev L Cheng H Goel S Perez-Soler R Impact and diagnostic gaps of comprehensive genomic profiling in real-world clinical practice. *Cancers.* (2020) 12:1156. 10.3390/cancers12051156 32375398 PMC7281757

[21] Chawla A Janku F Wheler JJ Miller VA Ryan J Anhorn R Estimated cost of anticancer therapy directed by comprehensive genomic profiling in a single-center study. *JCO Precis Oncol.* (2018) 2:PO.18.00074. 10.1200/po.18.00074 32913996 PMC7446484

[22] Letai A. Functional precision cancer medicine-moving beyond pure genomics. *Nat Med.* (2017) 23:1028–35. 10.1038/nm.4389 28886003

[23] Lassalle S Hofman V Heeke S Benzaquen J Long E Poudenx M Targeted assessment of the EGFR status as reflex testing in treatment-naive non-squamous cell lung carcinoma patients: a single laboratory experience (LPCE, Nice, France). *Cancers.* (2020) 12:955. 10.3390/cancers12040955 32294880 PMC7225982

[24] Buono G Gerratana L Bulfoni M Provinciali N Basile D Giuliano M Circulating tumor DNA analysis in breast cancer: Is it ready for prime-time? *Cancer Treat Rev.* (2019) 73:73–83. 10.1016/j.ctrv.2019.01.004 30682661

[25] Sedlackova T Repiska G Celec P Szemes T Minarik G. Fragmentation of DNA affects the accuracy of the DNA quantitation by the commonly used methods. *Biol Proced Online.* (2013) 15:5. 10.1186/1480-9222-15-5 23406353 PMC3576356

[26] Thakur BK Zhang H Becker A Matei I Huang Y Costa-Silva B Double-stranded DNA in exosomes: a novel biomarker in cancer detection. *Cell Res.* (2014) 24:766–9. 10.1038/cr.2014.44 24710597 PMC4042169

[27] Chen M Zhao H. Next-generation sequencing in liquid biopsy: cancer screening and early detection. *Hum Genomics.* (2019) 13:34. 10.1186/s40246-019-0220-8 31370908 PMC6669976

[28] Rangachari D Drake L Huberman MS McDonald DC VanderLaan PA Folch E Rapidly fatal advanced EGFR-mutated lung cancers and the need for rapid tumor genotyping in clinical practice. *Cancer Treat Commun.* (2016) 9:41–3. 10.1016/j.ctarc.2016.07.001 28111612 PMC5241076

[29] Ossandon MR Agrawal L Bernhard EJ Conley BA Dey SM Divi RL Circulating tumor DNA assays in clinical cancer research. *J Natl Cancer Inst.* (2018) 110:929–34. 10.1093/jnci/djy105 29931312 PMC6136923

[30] Schwaederle M Daniels GA Piccioni DE Fanta PT Schwab RB Shimabukuro KA On the road to precision cancer medicine: analysis of genomic biomarker actionability in 439 patients. *Mol Cancer Ther.* (2015) 14:1488–94. 10.1158/1535-7163.mct-14-1061 25852059

