# Supplementary figures and images for: From tumor mutational burden to characteristic targets analysis: Identifying the predictive biomarkers and natural product interventions in cancer management
Source: Front Nutr. 2022 Sep 20;9:989989. doi: 10.3389/fnut.2022.989989 (PMC9530334; doi:10.3389/fnut.2022.989989)

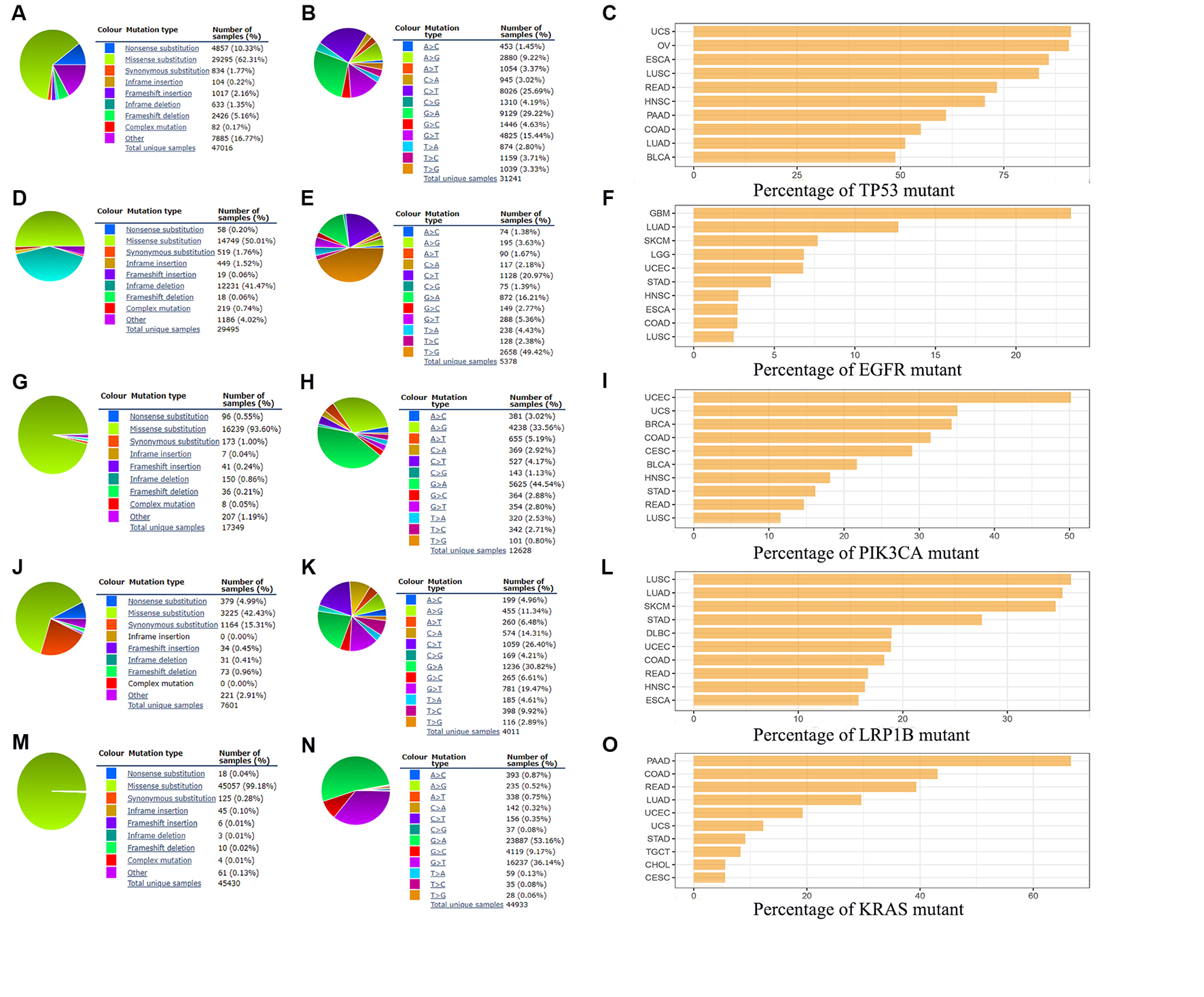

Supplement: Supplementary Figure S1 — An overview of the types of mutation and breakdown of the substitution mutations at the five most common mutation sites in this study. (A–C) TP53; (D–F) EGFR; (G–I) PIK3CA; (J–L) LRP1B; (M–O) KRAS. [file Image_1.JPEG]

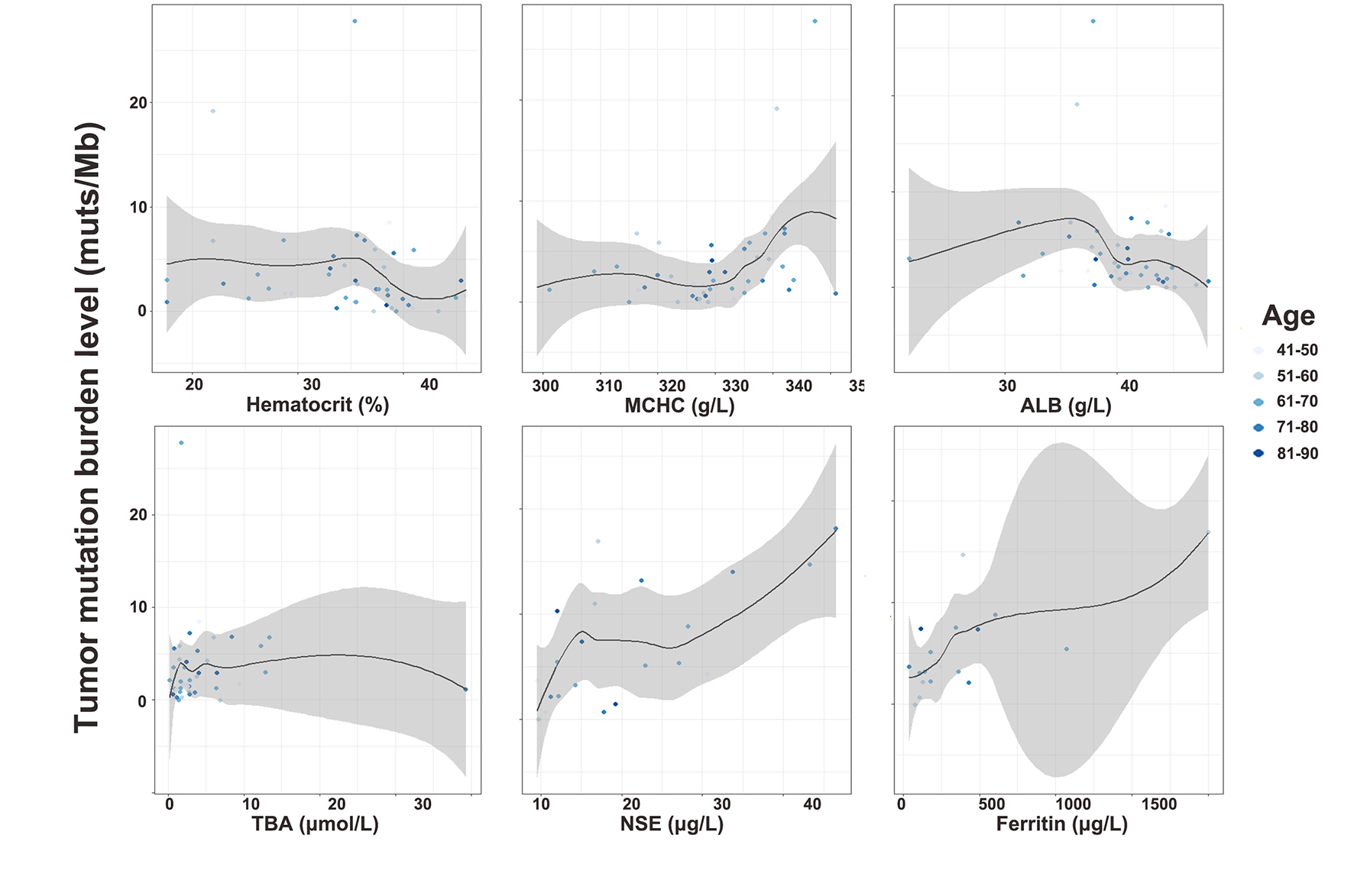

Supplement: Supplementary Figure S2 — Curve fitting correlation between TMB and clinical indicators. Unadjusted data is fitted according to locally weighted scatterplot smoothing, showing the trend of association between data. Especially with the increase of NSE and ferritin, the level of TMB increased significantly. MCHC, mean corpuscular hemoglobin concentration; ALB, albumin; TBA, total bile acid; NSE, neuron-specific enolase. [file Image_2.JPEG]

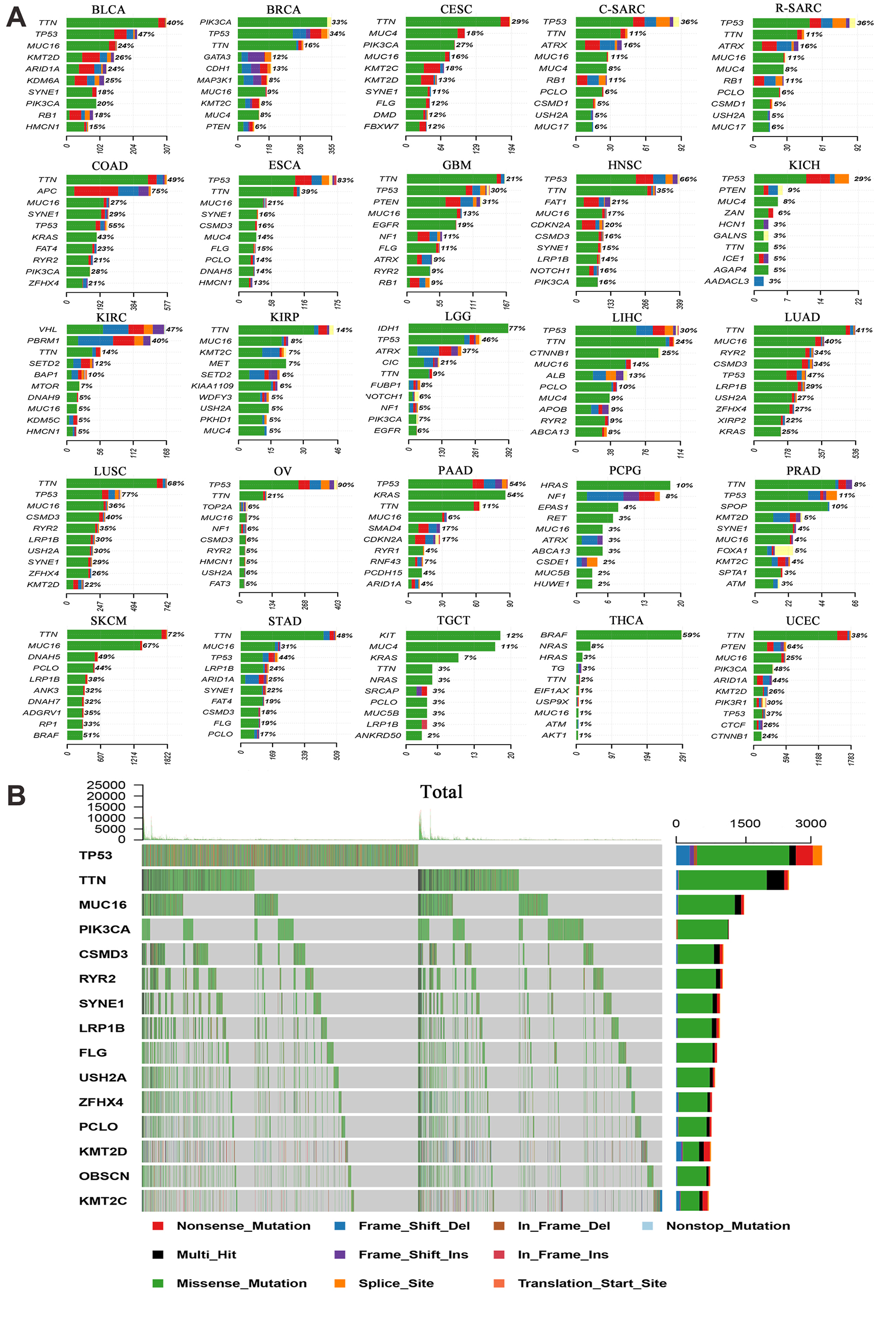

Supplement: Supplementary Figure S3 — Analysis of tumor mutation characteristics based on TCGA Database. (A) Further analysis of the ten key sites with the highest mutation rate in each solid tumors (n = 25); (B) Mutation characteristics analysis of 9475 samples, the top 15 genes with the highest mutation frequency were displayed in waterfall map. [file Image_3.JPEG]

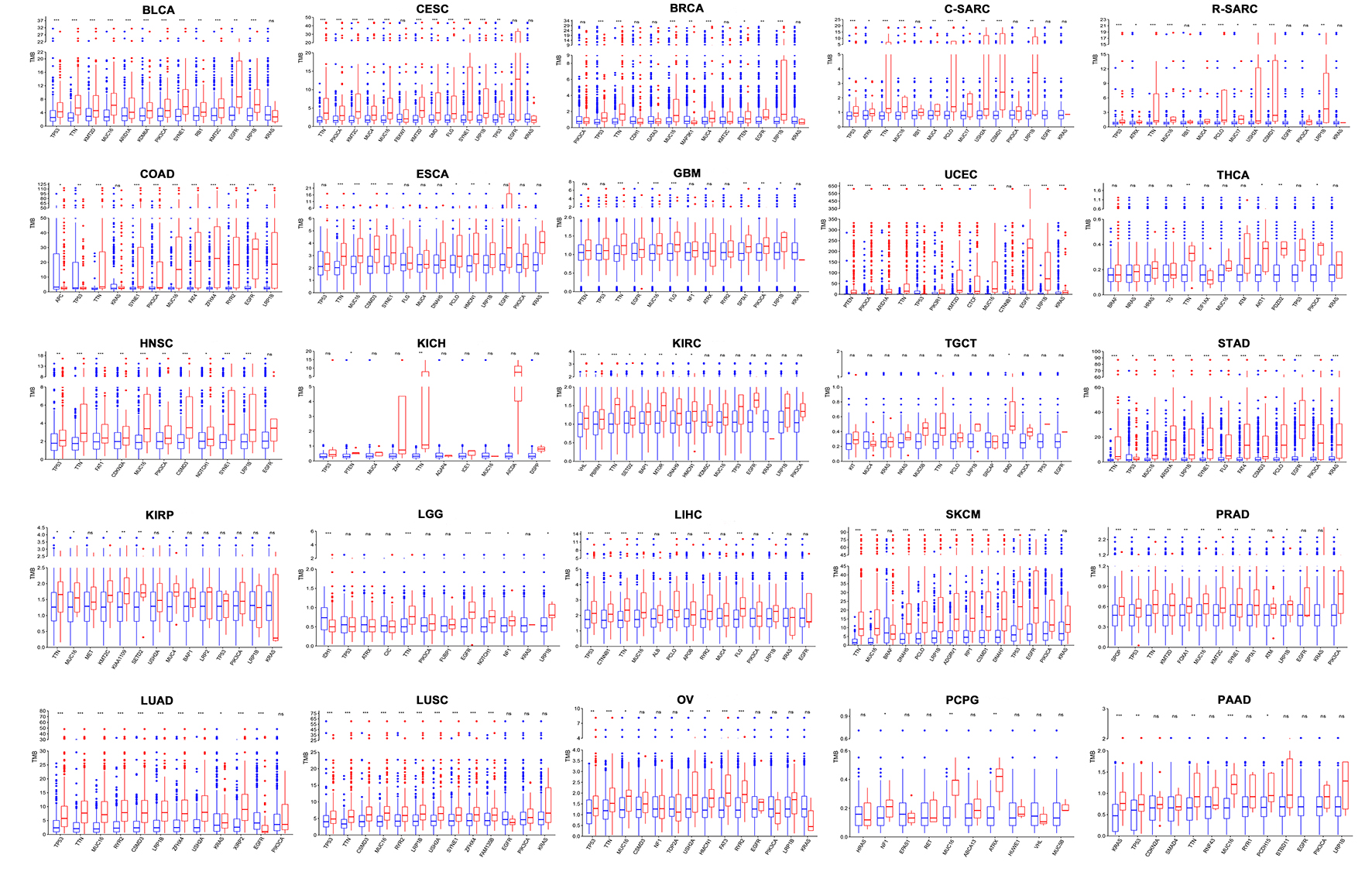

Supplement: Supplementary Figure S4 — The correlation between key targets (the top 10 sites with the highest mutation rate of 25 solid tumors and 5 key mutation sites identified in clinical samples) and TMB level in various tumors. [file Image_4.JPEG]

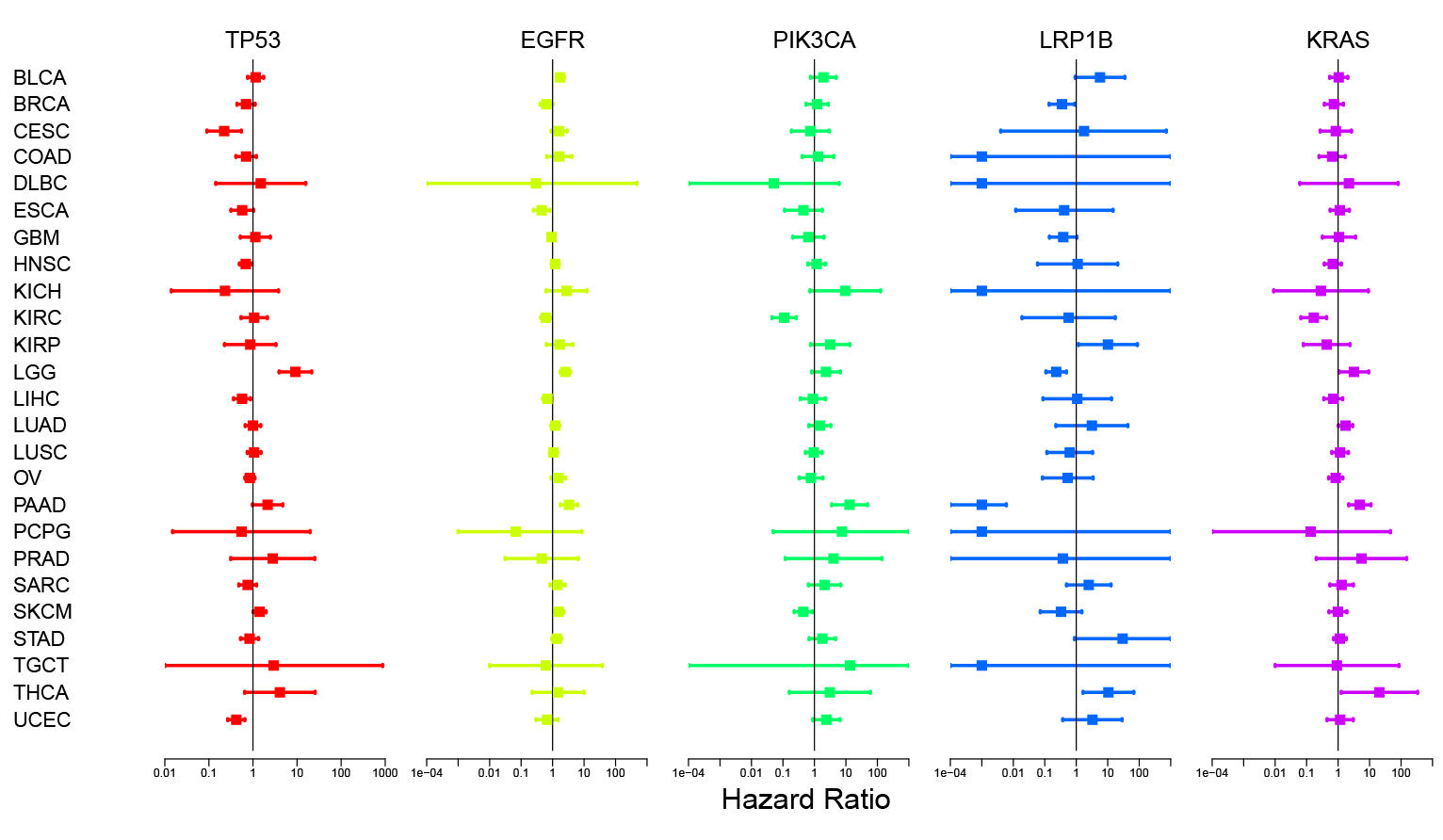

Supplement: Supplementary Figure S5 — Survival risk analysis of 5 key targets, risk ratios were calculated based on expression and clinical information. The primary sarcomas in retroperitoneum, peritoneum, connective, subcutaneous and other soft tissues are summarized and collectively referred to as SARC. [file Image_5.JPEG]

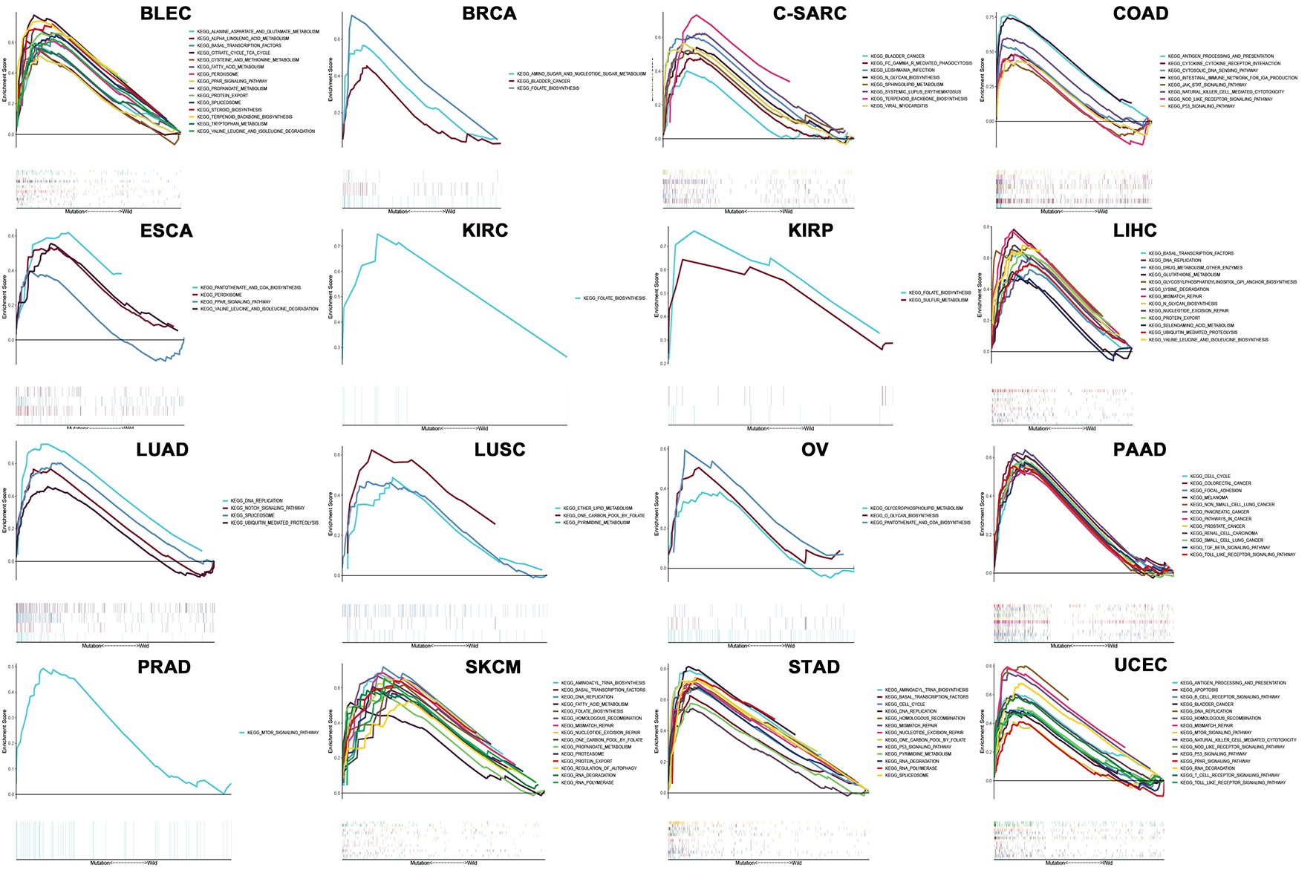

Supplement: Supplementary Figure S6 — KEGG derived gene sets were used to implement GSEA, which focused on single gene-LRP1B for the phenotype. [file Image_6.JPEG]
