# Supplementary material for: From tumor mutational burden to characteristic targets analysis: Identifying the predictive biomarkers and natural product interventions in cancer management
Source: Front Nutr. 2022 Sep 20;9:989989. doi: 10.3389/fnut.2022.989989 (PMC9530334; doi:10.3389/fnut.2022.989989)
Supplement: Supplementary file 7 [file Table_1.DOCX]

| Table S1 Primary patient diagnoses (n = 177) | | | |
| --- | --- | --- | --- |
| Type of tumor | Number | Type of tumor | Number |
| Non small cell Lung cancer | 56 | Esophageal cancer | 10 |
| Liver cancer | 6 | Pancreatic cancer | 5 |
| Colorectal cancer | 18 | Cholangio carcinoma | 4 |
| Breast cancer | 11 | Desmoplastic small round cell tumor | 1 |
| Gastric cancer | 17 | submandibular gland carcinoma | 1 |
| Cervical cancer | 3 | Oral cancer (including tongue cancer, laryngeal carcinoma, gingival cancer,carcinoma in the floor of the mouth and buccal cancer) | 7 |
| Ovarian cancer | 6 | Thyroid cancer | 1 |
| Small cell lung cancer | 8 | Renal cancer | 3 |
| Bladder cancer | 2 | Adrenal carcinoma | 3 |
| Parotid cancer | 1 | Thymoma | 1 |
| Endometrial cancer | 1 | Nasopharyngeal carcinoma | 1 |
| Neuroendocrine tumor | 1 | Gastrointestinal stromal tumor | 1 |
| Sarcoma | 9 |  |  |
