# Supplementary material for: From tumor mutational burden to characteristic targets analysis: Identifying the predictive biomarkers and natural product interventions in cancer management
Source: Front Nutr. 2022 Sep 20;9:989989. doi: 10.3389/fnut.2022.989989 (PMC9530334; doi:10.3389/fnut.2022.989989)
Supplement: Supplementary file 8 [file Table_2.DOC]

| Table S2 Evaluation of the curative effect and comparison of medical costs after NGS-guided alterations to the clinical management plan | | | | | | |
| --- | --- | --- | --- | --- | --- | --- |
| Patient | Type of Tumor | Last line chemotherapy | Management change | Observed Benefit (CR + PR + SD) | Daily treatment cost | |
|  |  |  |  |  | Chemotherapy | Targeted Therapy |
| patient 1 | Lung cancer | Etoposide + Nedaplatin | Toripalimab + Etoposide + Cisplatin | Yes | 1247.3 | 1341.6 |
| patient 3 | Gingival cancer | Paclitaxel-albumin | Toripalimab | Yes | 672.8 | 756.7 |
| patient 4 | Lung cancer | Gemcitabine + Carboplatin | Almonertinib | Yes | 1504.85 | 902.8 |
| patient 9 | Mediastinal cancer; Adrenal cancer | Pemetrexed + Carboplatin | Toripalimab + Gemcitabine | Yes | 1240.3 | 820.4 |
| patient 11 | Bladder cancer | Docetaxe + Nedaplatin | Apatinib | NA | - | - |
| patient 16 | Parotid gland cancer | NA | Toripalimab | NA | - | - |
| patient 19 | Lung cancer | NA | Toripalimab | No | - | - |
| patient 20 | Submandibular gland cancer | Gemcitabine + Fluorouracil + Cisplatin | Sintilimab + Doxorubicin + Cyclophosphamide | Yes | 2025.31 | 1024.02 |
| patient 22 | Tongue cancer | Etoposide + Cisplatin | Anlotinib | Yes | 844.5 | 925.8 |
| patient 25 | Lung cancer | NA | Gefitinib + Pemetrexed + Cisplatin | Yes | - | - |
| patient 27 | Lung cancer | Pemetrexed + Carboplatin | Crizotinib | Yes | 762.09 | 703.63 |
| patient 29 | Colon cancer | Oxaliplatin + Capecitabine | Sintilimab + Irinotecan + Capecitabine | No | 1072.56 | 1216.38 |
| patient 31 | Lung cancer | Docetaxe + Capecitabine | Toripalimab + Docetaxe + Carboplatin | Yes | 749.65 | 2760.26 |
| patient 32 | Rectal cancer | Irinotecan + Calcium folinate + Fluorouracil | Camrelizumab + Regorafenib | No | - | - |
| patient 34 | Lung cancer | Etoposide + Cisplatin | Sintilimab + Irinotecan + Nedaplatin | Yes | 1265.47 | 1998.96 |
| patient 36 | Lung cancer | Paclitaxel + Nedaplatin | Toripalimab | NA | - | - |
| patient 37 | Lung cancer | Irinotecan + Carboplatin | Sintilimab | Yes | 1061.8 | 1381.32 |
| patient 44 | Lung cancer | Etoposide + Cisplatin | Sintilimab + Etoposide + Cisplatin | No | - | - |
| patient 49 | Rectal cancer | NA | Sintilimab | No | - | - |
| patient 51 | Esophageal cancer | Paclitaxel + Cisplatin | Sintilimab | Yes | - | - |
| patient 52 | Esophageal cancer | Irinotecan + Nedaplatin | Sintilimab + Paclitaxel-albumin + Nedaplatin | Yes | 1102.13 | 1695.04 |
| patient 53 | Cervical cancer | Fluorouracil + Epirubicin + Cyclophosphamide | Sintilimab + Fluorouracil + Epirubicin + Cyclophosphamide | Yes | 1137.8 | 1896.74 |
| patient 54 | Lung cancer | Pemetrexed + Carboplatin | Gefitinib | Yes | 1299.89 | 980.19 |
| patient 124 | Lung cancer | Etoposide + Cisplatin | Sintilimab + Etoposide + Cisplatin | Yes | 1149.04 | 1278.36 |
