# Supplementary material for: From tumor mutational burden to characteristic targets analysis: Identifying the predictive biomarkers and natural product interventions in cancer management
Source: Front Nutr. 2022 Sep 20;9:989989. doi: 10.3389/fnut.2022.989989 (PMC9530334; doi:10.3389/fnut.2022.989989)
Supplement: Supplementary file 9 [file Table_3.DOCX]

| Table S3 Genomic features of 25 solid tumors from TCGA database | | | | | | | | |
| --- | --- | --- | --- | --- | --- | --- | --- | --- |
| Tumor type | Transcriptome samples  (Nomal / Tumor) | Mutant samples | No.TP53 | No.EGFR | No.PIK3CA | No.LRP1B | No.KRAS | TOP 10 mutation gene frequency (symbol / Number) |
| LUAD | 594 (59/535) | 522 | 249 | 68 | 23 | 157 | 138 | TP53/249 TTN/229 MUC16/223 RYR2/188 CSMD3/185 LRP1B/157 ZFHX4/154 USH2A/149 KRAS/138 XIRP2/126 |
| LUSC | 551 (49/502) | 489 | 350 | 7 | 48 | 143 | 7 | TP53/350 TTN/330 CSMD3/190 MUC16/177 RYR2/171 LRP1B/143 USH2A/142 SYNE1/141 ZFHX4/130 FAM135B/106 |
| BRCA | 1222 (113/1109) | 980 | 314 | 10 | 322 | 26 | 6 | PIK3CA/322 TP53/314 TTN/156 CDH1/114 GATA3/99 MUC16/84 MAP3K1/80 MUC4/74 KMT2C/72 PTEN/51 |
| COAD | 514 (41/473) | 399 | 214 | 10 | 113 | 71 | 170 | APC/297 TP53/214 TTN/193 KRAS/170 SYNE1/116 PIK3CA/113 MUC16/108 FAT4/93 ZFHX4/84 RYR2/82 |
| OV | 379 (0/379) | 436 | 359 | 5 | 6 | 18 | 5 | TP53/359 TTN/93 MUC16/28 CSMD3/27 NF1/26 TOP2A/26 USH2A/24 HMCN1/23 FAT3/22 RYR2/22 |
| KIRC | 611 (72/539) | 336 | 5 | 2 | 4 | 8 | 1 | VHL/143 PBRM1/122 TTN/47 SETD2/37 BAP1/30 MTOR/22 DNAH9/17 HMCN1/17 KDM5C/17 MUC16/17 |
| KIRP | 321 (32/289) | 281 | 6 | 0 | 3 | 6 | 5 | TTN/38 MUC16/22 MET/21 KMT2C/19 KIAA1109/17 SETD2/17 USH2A/14 MUC4/13 BAP1/12 LRP2/12 |
| KICH | 89 (24/65) | 66 | 16 | 0 | 0 | 0 | 0 | TP53/16 PTEN/6 MUC4/5 ZAN/4 TTN/3 AGAP4/3 ICE1/3 MUC16/2 AICDA/2 DSPP/2 |
| SKCM | 472 (1/471) | 466 | 60 | 29 | 10 | 175 | 10 | TTN/332 MUC16/312 BRAF/236 DNAH5/227 PCLO/206 LRP1B/175 ADGRV1/161 RP1/155 CSMD1/150 DNAH7/149 |
| PAAD | 182 (4/178) | 149 | 82 | 1 | 4 | 5 | 85 | KRAS/85 TP53/82 CDKN2A/27 SMAD4/27 TTN/17 RNF43/10 MUC16/10 RYR1/7 PCDH15/7 BTBD11/6 |
| PRAD | 551 (52/499) | 475 | 45 | 3 | 9 | 13 | 2 | SPOP/49 TP53/45 TTN/41 KMT2D/24 FOXA1/22 MUC16/20 KMT2C/19 SYNE1/17 SPTA1/16 ATM/16 |
| BLCA | 423 (19/414) | 411 | 190 | 6 | 84 | 38 | 16 | TP53/190 TTN/161 KMT2D/104 MUC16/98 ARID1A/97 KDM6A/90 PIK3CA/84 SYNE1/73 RB1/60 KMT2C/58 |
| LIHC | 424 (50/374) | 361 | 100 | 5 | 10 | 28 | 5 | TP53/100 CTNNB1/91 TTN/85 MUC16/52 ALB/40 PCLO/36 APOB/34 RYR2/31 MUC4/31 FLG/29 |
| C-SARC | 120（1/119） | 237 | 74 | 1 | 6 | 10 | 1 | TP53/74 ATRX/35 TTN/27 MUC16/25 RB1/20 MUC4/18 PCLO/15 MUC17/14 USH2A/13 CSMD1/12 |
| THCA | 568 (58/510) | 483 | 2 | 0 | 3 | 0 | 4 | BRAF/289 NRAS/39 HRAS/17 TG/15 TTN/10 EIF1AX/6 MUC16/6 ATM/6 AKT1/5 PDZD2/5 |
| GBM | 117 (3/114) | 389 | 111 | 74 | 32 | 12 | 2 | PTEN/115 TP53/111 TTN/80 EGFR/74 MUC16/51 FLG/42 NF1/39 ATRX/34 RYR2/34 SPTA1/33 |
| LGG | 529 (0/529) | 503 | 226 | 30 | 35 | 6 | 1 | IDH1/390 TP53/226 ATRX/177 CIC/102 TTN/47 PIK3CA/35 FUBP1/35 EGFR/30 NOTCH1/29 NF1/25 |
| ESCA | 171 (11/160) | 183 | 141 | 5 | 15 | 20 | 2 | TP53/141 TTN/72 MUC16/38 CSMD3/29 SYNE1/28 FLG/27 MUC4/26 DNAH5/26 PCLO/25 HMCN1/24 |
| STAD | 407 (32/375) | 431 | 176 | 17 | 63 | 102 | 34 | TTN/208 TP53/176 MUC16/133 ARID1A/103 LRP1B/102 SYNE1/93 FLG/84 FAT4/82 CSMD3/79 PCLO/72 |
| CESC | 309 (3/306) | 288 | 22 | 6 | 77 | 32 | 16 | TTN/83 PIK3CA/77 KMT2C/53 MUC4/51 MUC16/46 FBXW7/36 KMT2D/36 DMD/35 FLG/35 SYNE1/33 |
| PCPG | 153 (3/150) | 175 | 0 | 0 | 0 | 0 | 0 | HRAS/18 NF1/14 EPAS1/8 RET/6 MUC16/5 ABCA13/5 ATRX/5 HUWE1/3 VHL/3 MUC5B/3 |
| UCEC | 575 (23/552) | 529 | 190 | 32 | 254 | 90 | 96 | PTEN/331 PIK3CA/254 ARID1A/232 TTN/201 TP53/190 PIK3R1/148 KMT2D/140 CTCF/131 MUC16/129 CTNNB1/128 |
| TGCT | 156 (0/156) | 143 | 1 | 1 | 3 | 4 | 10 | KIT/17 MUC4/16 KRAS/10 NRAS/5 MUC5B/4 TTN/4 PCLO/4 LRP1B/4 SRCAP/4 DMD/3 |
| R-SARC | 100 (1/99) | 237 | 74 | 1 | 6 | 10 | 1 | TP53/74 ATRX/35 TTN/27 MUC16/25 RB1/20 MUC4/18 PCLO/15 MUC17/14 USH2A/13 CSMD1/12 |
| HNSC | 123 (12/111) | 506 | 313 | 9 | 81 | 69 | 0 | TP53/313 TTN/175 FAT1/104 CDKN2A/89 MUC16/85 PIK3CA/81 CSMD3/81 NOTCH1/78 SYNE1/75 LRP1B/69 |
