# Supplementary material for: From tumor mutational burden to characteristic targets analysis: Identifying the predictive biomarkers and natural product interventions in cancer management
Source: Front Nutr. 2022 Sep 20;9:989989. doi: 10.3389/fnut.2022.989989 (PMC9530334; doi:10.3389/fnut.2022.989989)
Supplement: Supplementary file 10 [file Table_4.DOCX]

| Table S4 The survival analysis of the key mutant genes in 25 solid tumors from TCGA database | | | | | | |
| --- | --- | --- | --- | --- | --- | --- |
| Tumor type | p-value: TP53 | p-value: EGFR | p-value: PIK3CA | p-value: LRP1B | p-value: KRAS | TOP10 mutation gene  (symbol / p-value) |
| LUAD | 0.165 | 0.128 | 0.642 | 0.852 | 0.639 | TP53/0.165 ZFHX4/0.261 RYR2/0.423 XIRP2/0.576 KRAS/0.639 CSMD3/0.685 LRP1B/0.852 USH2A/0.859 TTN/0.865 MUC16/0.956 |
| LUSC | 0.203 | 0.547 | 0.459 | 0.878 | 0.351 | TTN/0.0008 RYR2/0.044 CSMD3/0.072 FAM135B/0.100 SYNE1/0.162 TP53/0.203 ZFHX4/0.332 MUC16/0.832 LRP1B/0.878 USH2A/0.977 |
| BRCA | 0.121 | 0.443 | 0.686 | 0.389 | 0.754 | TP53/0.121 KMT2C/0.138 TTN/0.199 PTEN/0.233 GATA3/0.326 CDH1/0.445 MUC16/0.560 PIK3CA/0.686 MAP3K1/0.752 MUC4/0.767 |
| COAD | 0.415 | 0.021 | 0.863 | 0.656 | 0.842 | SYNE1/0.144 TTN/0.173 RYR2/0.233 ZFHX4/0.368 APC/0.381 TP53/0.415 KRAS/0.842 PIK3CA/0.863 MUC16/0.869 FAT4/0.932 |
| OV | 0.162 | 0.266 | 0.170 | 0.200 | 0.845 | CSMD3/0.010 TOP2A/0.016 TTN/0.086 HMCN1/0.115 TP53/0.162 FAT3/0.194 RYR2/0.262 MUC16/0.457 USH2A/0.664 NF1/0.796 |
| KIRC | 0.024 | 0.374 | 0.910 | 0.988 | 0.555 | BAP1/0.002 HMCN1/0.003 KDM5C/0.195 MTOR/0.496 VHL/0.533 MUC16/0.543 PBRM1/0.543 SETD2/0.567 TTN/0.643 DNAH9/0.795 |
| KIRP | 0.162 | NA | 0.337 | 0.585 | 0.475 | SETD2/0.002 TTN/0.288 MUC16/0.431 KIAA1109/0.454 LRP2/0.481 USH2A/0.605 MUC4/0.795 KMT2C/0.810 BAP1/0.845 MET/0.896 |
| KICH | 0.034 | NA | NA | NA | NA | PTEN/0.009 ZAN/0.029 TP53/0.034 TTN/0.247 AICDA/0.247 AGAP4/0.456 ICE1/0.482 MUC16/0.596 MUC4/0.660 DSPP/0.691 |
| SKCM | 0.449 | 0.172 | 0.689 | 0.467 | 0.233 | MUC16/0.001 ADGRV1/0.010 CSMD1/0.086 TTN/0.091 BRAF/0.115 RP1/0.124 DNAH5/0.183 PCLO/0.234 DNAH7/0.369 LRP1B/0.467 |
| PAAD | 0.002 | 0.664 | 0.679 | 0.615 | 0.002 | KRAS/0.002 TP53/0.002 CDKN2A/0.004 MUC16/0.124 BTBD11/0.203 PCDH15/0.427 RNF43/0.461 RYR1/0.525 SMAD4/0.550 TTN/0.718 |
| PRAD | 0.908 | 0.759 | 0.481 | 0.239 | 0.858 | KMT2C/0.027 ATM/0.136 TTN/0.310 MUC16/0.489 KMT2D/0.510 FOXA1/0.564 SPTA1/0.592 SYNE1/0.638 SPOP/0.873 TP53/0.908 |
| BLCA | 0.912 | 0.513 | 0.286 | 0.125 | 0.002 | RB1/0.043 KMT2D/0.082 SYNE1/0.110 MUC16/0.185 PIK3CA/0.286 TTN/0.390 KMT2C/0.816 KDM6A/0.870 TP53/0.912 ARID1A/0.991 |
| LIHC | 0.035 | 0.474 | 0.456 | 0.010 | 0.527 | TP53/0.035 MUC16/0.088 APOB/0.162 RYR2/0.271 PCLO/0.271 MUC4/0.315 TTN/0.488 FLG/0.496 CTNNB1/0.579 ALB/0.832 |
| C-SARC | 0.782 | 0.778 | 0.353 | 0.469 | NA | TP53/0.782 ATRX/0.075 TTN/0.929 MUC16/0.169 RB1/0.784 MUC4/0.126 PCLO/0.534 MUC17/0.655 USH2A/0.763 CSMD1/0.340 |
| THCA | 0.807 | NA | 0.001 | NA | 0.878 | EIF1AX/0.001 ATM/0.082 TG/0.350 NRAS/0.393 TTN/0.512 HRAS/0.539 MUC16/0.720 BRAF/0.754 PDZD2/0.790 AKT1/0.829 |
| GBM | 0.007 | 0.701 | 0.145 | 0.736 | 0.849 | TP53/0.007 ATRX/0.017 MUC16/0.053 FLG/0.306 TTN/0.426 RYR2/0.461 PTEN/0.513 SPTA1/0.547 EGFR/0.701 NF1/0.845 |
| LGG | 0.075 | 0.000 | 0.061 | 0.445 | 0.499 | IDH1/0.000 EGFR/0.000 CIC/0.000 NF1/0.000 PIK3CA/0.061 TP53/0.075 FUBP1/0.136 ATRX/0.156 NOTCH1/0.166 TTN/0.220 |
| ESCA | 0.279 | 0.965 | 0.630 | 0.464 | 0.782 | TTN/0.095 SYNE1/0.173 TP53/0.279 FLG/0.450 PCLO/0.554 MUC16/0.614 MUC4/0.669 HMCN1/0.765 CSMD3/0.924 DNAH5/0.957 |
| STAD | 0.272 | 0.863 | 0.085 | 0.164 | 0.340 | FAT4/0.014 MUC16/0.014 FLG/0.033 TTN/0.051 ARID1A/0.162 LRP1B/0.164 TP53/0.272 PCLO/0.290 CSMD3/0.452 SYNE1/0.617 |
| CESC | 0.297 | 0.928 | 0.723 | 0.560 | 0.154 | SYNE1/0.058 TTN/0.077 KMT2D/0.196 FBXW7/0.252 KMT2C/0.395 MUC4/0.447 DMD/0.589 FLG/0.606 PIK3CA/0.723 MUC16/0.741 |
| PCPG | NA | NA | NA | NA | NA | ATRX/0.000 MUC16/0.000 HRAS/0.455 NF1/0.574 ABCA13/0.650 EPAS1/0.700 RET/0.710 MUC5B/0.770 HUWE1/0.794 VHL/0.822 |
| UCEC | 0.000 | 0.037 | 0.035 | 0.011 | 0.042 | ARID1A/0.000 PTEN/0.000 TP53/0.000 MUC16/0.006 TTN/0.013 PIK3CA/0.035 CTCFL/0.042 CTNNB1/0.067 KMT2D/0.105 PIK3R1/0.302 |
| TGCT | 0.928 | 0.867 | 0.770 | 0.770 | 0.113 | KRAS/0.113 KIT/0.381 MUC4/0.589 TTN/0.735 PCLO/0.747 SRCAP/0.747 MUC5B/0.759 LRP1B/0.770 DMD/0.770 NRAS/0.775 |
| R-SARC | 0.327 | NA | NA | NA | NA | MUC4/0.000 CSMD1/0.057 TTN/0.119 RB1/0.293 PCLO/0.316 TP53/0.327 ATRX/0.423 MUC17/0.597 MUC16/0.707 USH2A/0.869 |
| HNSC | 0.071 | 0.810 | 0.629 | 0.340 | NA | NOTCH1/0.002 TP53/0.071 LRP1B/0.340 SYNE1/0.357 TTN/0.455 FAT1/0.611 PIK3CA/0.629 CSMD3/0.817 CDKN2A/0.901 MUC16/0.987 |
