# Supplementary material for: From tumor mutational burden to characteristic targets analysis: Identifying the predictive biomarkers and natural product interventions in cancer management
Source: Front Nutr. 2022 Sep 20;9:989989. doi: 10.3389/fnut.2022.989989 (PMC9530334; doi:10.3389/fnut.2022.989989)
Supplement: Supplementary file 11 [file Table_5.DOCX]

| Table S5 LRP1B mutations in 1683 patients with pan-cancer | | | | |
| --- | --- | --- | --- | --- |
| Type of tumor | Number of  LRP1B mutant type patients | Number of  LRP1B wild-type patients | Sample size | Mutation rate (%) |
| Non-small cell lung cancer | 111 | 763 | 874 | 12.7 |
| colorectal cancer | 51 | 192 | 243 | 21.0 |
| Gastric cancer | 34 | 92 | 126 | 27.0 |
| Breast cancer | 8 | 110 | 118 | 6.8 |
| Cholangiocarcinoma | 5 | 47 | 52 | 9.6 |
| Pancreatic cancer | 6 | 36 | 42 | 14.3 |
| Ovarian cancer | 6 | 25 | 31 | 19.4 |
| Liver cancer | 1 | 29 | 30 | 3.3 |
| Small cell lung cancer | 7 | 17 | 24 | 29.2 |
| Melanoma | 2 | 22 | 24 | 8.3 |
| Soft tissue sarcoma | 5 | 19 | 24 | 20.8 |
| Esophagus cancer | 3 | 17 | 20 | 15.0 |
| Urothelial carcinoma | 2 | 14 | 16 | 12.5 |
| Cervical cancer | 4 | 11 | 15 | 26.7 |
| Small intestinal carcinoma | 3 | 12 | 15 | 20.0 |
| Head and neck cancer | 2 | 13 | 15 | 13.3 |
| Prostatic cancer | 5 | 9 | 14 | 35.7 |
| Total | 255 | 1428 | 1683 | 15.2 |
