# Supplementary material for: From tumor mutational burden to characteristic targets analysis: Identifying the predictive biomarkers and natural product interventions in cancer management
Source: Front Nutr. 2022 Sep 20;9:989989. doi: 10.3389/fnut.2022.989989 (PMC9530334; doi:10.3389/fnut.2022.989989)
Supplement: Supplementary file 12 [file Table_6.DOCX]

| Table S6 Kegg enrichment analysis with a single gene-LRP1B for the phenotype based on GSEA | | | |
| --- | --- | --- | --- |
| Tumor type | Pathway | Normalized enrichment score | p-value |
| LUAD | KEGG_RNA_DEGRADATION | 1.92 | 0.004 |
|  | KEGG_CELL_CYCLE | 1.86 | 0.006 |
|  | KEGG_BASAL_TRANSCRIPTION_FACTORS | 1.84 | 0.009 |
|  | KEGG_NOTCH_SIGNALING_PATHWAY | 1.83 | 0.006 |
|  | KEGG_SPLICEOSOME | 1.81 | 0.028 |
|  | KEGG_OOCYTE_MEIOSIS | 1.8 | 0.016 |
|  | KEGG_UBIQUITIN_MEDIATED_PROTEOLYSIS | 1.8 | 0.004 |
|  | KEGG_PATHOGENIC_ESCHERICHIA_COLI_INFECTION | 1.74 | 0.019 |
|  | KEGG_PROTEASOME | 1.68 | 0.054 |
|  | KEGG_HOMOLOGOUS_RECOMBINATION | 1.65 | 0.04 |
|  | KEGG_PROGESTERONE_MEDIATED_OOCYTE_MATURATION | 1.64 | 0.026 |
|  | KEGG_PYRIMIDINE_METABOLISM | 1.64 | 0.035 |
|  | KEGG_DNA_REPLICATION | 1.63 | 0.046 |
|  | KEGG_MISMATCH_REPAIR | 1.59 | 0.061 |
|  | KEGG_GLYOXYLATE_AND_DICARBOXYLATE_METABOLISM | 1.59 | 0.038 |
|  | KEGG_RNA_POLYMERASE | 1.58 | 0.046 |
| LUSC | KEGG_ONE_CARBON_POOL_BY_FOLATE | 1.67 | 0.037 |
|  | KEGG_PYRIMIDINE_METABOLISM | 1.6 | 0.049 |
|  | KEGG_ETHER_LIPID_METABOLISM | 1.58 | 0.026 |
| BRCA | KEGG_FOLATE_BIOSYNTHESIS | 1.78 | 0 |
|  | KEGG_AMINO_SUGAR_AND_NUCLEOTIDE_SUGAR_METABOLISM | 1.69 | 0.011 |
|  | KEGG_BLADDER_CANCER | 1.49 | 0.038 |
| COAD | KEGG_ANTIGEN_PROCESSING_AND_PRESENTATION | 2.17 | 0 |
|  | KEGG_NATURAL_KILLER_CELL_MEDIATED_CYTOTOXICITY | 2.05 | 0.002 |
|  | KEGG_AUTOIMMUNE_THYROID_DISEASE | 1.97 | 0 |
|  | KEGG_GRAFT_VERSUS_HOST_DISEASE | 1.94 | 0.002 |
|  | KEGG_CYTOSOLIC_DNA_SENSING_PATHWAY | 1.93 | 0.006 |
|  | KEGG_ASTHMA | 1.93 | 0 |
|  | KEGG_INTESTINAL_IMMUNE_NETWORK_FOR_IGA_PRODUCTION | 1.9 | 0.002 |
|  | KEGG_ALLOGRAFT_REJECTION | 1.88 | 0.006 |
|  | KEGG_HEMATOPOIETIC_CELL_LINEAGE | 1.78 | 0.024 |
|  | KEGG_TYPE_I_DIABETES_MELLITUS | 1.76 | 0.026 |
|  | KEGG_VIRAL_MYOCARDITIS | 1.75 | 0.022 |
|  | KEGG_CYTOKINE_CYTOKINE_RECEPTOR_INTERACTION | 1.7 | 0.027 |
|  | KEGG_PROTEASOME | 1.67 | 0.043 |
|  | KEGG_NOD_LIKE_RECEPTOR_SIGNALING_PATHWAY | 1.61 | 0.048 |
|  | KEGG_JAK_STAT_SIGNALING_PATHWAY | 1.58 | 0.042 |
|  | KEGG_P53_SIGNALING_PATHWAY | 1.56 | 0.045 |
| OV | KEGG_PANTOTHENATE_AND_COA_BIOSYNTHESIS | 1.67 | 0.012 |
|  | KEGG_O_GLYCAN_BIOSYNTHESIS | 1.54 | 0.04 |
|  | KEGG_GLYCEROPHOSPHOLIPID_METABOLISM | 1.4 | 0.035 |
| KIRC | KEGG_FOLATE_BIOSYNTHESIS | 1.69 | 0.011 |
| KIRP | KEGG_FOLATE_BIOSYNTHESIS | 1.72 | 0.011 |
|  | KEGG_SULFUR_METABOLISM | 1.6 | 0.01 |
| KICH | NA | NA | NA |
| SKCM | KEGG_PYRIMIDINE_METABOLISM | 2.05 | 0 |
|  | KEGG_RNA_POLYMERASE | 2.02 | 0 |
|  | KEGG_NUCLEOTIDE_EXCISION_REPAIR | 1.99 | 0.004 |
|  | KEGG_AMINOACYL_TRNA_BIOSYNTHESIS | 1.94 | 0.002 |
|  | KEGG_CYSTEINE_AND_METHIONINE_METABOLISM | 1.88 | 0 |
|  | KEGG_DNA_REPLICATION | 1.81 | 0.01 |
|  | KEGG_HOMOLOGOUS_RECOMBINATION | 1.8 | 0.012 |
|  | KEGG_GLYOXYLATE_AND_DICARBOXYLATE_METABOLISM | 1.78 | 0.01 |
|  | KEGG_RNA_DEGRADATION | 1.78 | 0.016 |
|  | KEGG_AMINO_SUGAR_AND_NUCLEOTIDE_SUGAR_METABOLISM | 1.76 | 0.021 |
|  | KEGG_MISMATCH_REPAIR | 1.75 | 0.016 |
|  | KEGG_RIBOFLAVIN_METABOLISM | 1.74 | 0.004 |
|  | KEGG_SPLICEOSOME | 1.74 | 0.034 |
|  | KEGG_BASE_EXCISION_REPAIR | 1.73 | 0.011 |
|  | KEGG_VALINE_LEUCINE_AND_ISOLEUCINE_BIOSYNTHESIS | 1.71 | 0.012 |
|  | KEGG_BASAL_TRANSCRIPTION_FACTORS | 1.69 | 0.018 |
|  | KEGG_SELENOAMINO_ACID_METABOLISM | 1.66 | 0.012 |
|  | KEGG_PROTEIN_EXPORT | 1.66 | 0.026 |
|  | KEGG_ONE_CARBON_POOL_BY_FOLATE | 1.65 | 0.021 |
|  | KEGG_PROTEASOME | 1.65 | 0.049 |
|  | KEGG_PROPANOATE_METABOLISM | 1.65 | 0.045 |
|  | KEGG_LYSINE_DEGRADATION | 1.64 | 0.04 |
|  | KEGG_CITRATE_CYCLE_TCA_CYCLE | 1.64 | 0.026 |
|  | KEGG_REGULATION_OF_AUTOPHAGY | 1.61 | 0.028 |
|  | KEGG_FOLATE_BIOSYNTHESIS | 1.6 | 0.031 |
|  | KEGG_PENTOSE_PHOSPHATE_PATHWAY | 1.6 | 0.033 |
|  | KEGG_PURINE_METABOLISM | 1.56 | 0.021 |
|  | KEGG_PEROXISOME | 1.56 | 0.049 |
|  | KEGG_NON_HOMOLOGOUS_END_JOINING | 1.51 | 0.048 |
|  | KEGG_FATTY_ACID_METABOLISM | 1.51 | 0.048 |
| PAAD | KEGG_PANCREATIC_CANCER | 1.78 | 0 |
|  | KEGG_LYSOSOME | 1.74 | 0 |
|  | KEGG_VIRAL_MYOCARDITIS | 1.74 | 0.003 |
|  | KEGG_COLORECTAL_CANCER | 1.73 | 0 |
|  | KEGG_RENAL_CELL_CARCINOMA | 1.71 | 0 |
|  | KEGG_LEISHMANIA_INFECTION | 1.69 | 0.012 |
|  | KEGG_PATHWAYS_IN_CANCER | 1.67 | 0 |
|  | KEGG_MELANOMA | 1.67 | 0 |
|  | KEGG_PRION_DISEASES | 1.65 | 0.01 |
|  | KEGG_REGULATION_OF_ACTIN_CYTOSKELETON | 1.63 | 0 |
|  | KEGG_PROSTATE_CANCER | 1.63 | 0.002 |
|  | KEGG_FC_GAMMA_R_MEDIATED_PHAGOCYTOSIS | 1.63 | 0 |
|  | KEGG_SMALL_CELL_LUNG_CANCER | 1.62 | 0.01 |
|  | KEGG_CELL_CYCLE | 1.61 | 0.028 |
|  | KEGG_GAP_JUNCTION | 1.6 | 0.006 |
|  | KEGG_TGF_BETA_SIGNALING_PATHWAY | 1.59 | 0.016 |
|  | KEGG_GLIOMA | 1.59 | 0.008 |
|  | KEGG_ENDOMETRIAL_CANCER | 1.58 | 0.012 |
|  | KEGG_FOCAL_ADHESION | 1.58 | 0.006 |
|  | KEGG_NATURAL_KILLER_CELL_MEDIATED_CYTOTOXICITY | 1.57 | 0.046 |
|  | KEGG_CYSTEINE_AND_METHIONINE_METABOLISM | 1.56 | 0.01 |
|  | KEGG_TOLL_LIKE_RECEPTOR_SIGNALING_PATHWAY | 1.54 | 0.039 |
|  | KEGG_PATHOGENIC_ESCHERICHIA_COLI_INFECTION | 1.54 | 0.022 |
|  | KEGG_PROGESTERONE_MEDIATED_OOCYTE_MATURATION | 1.53 | 0.021 |
|  | KEGG_N_GLYCAN_BIOSYNTHESIS | 1.53 | 0.035 |
|  | KEGG_SYSTEMIC_LUPUS_ERYTHEMATOSUS | 1.53 | 0.044 |
|  | KEGG_CHRONIC_MYELOID_LEUKEMIA | 1.52 | 0.009 |
|  | KEGG_UBIQUITIN_MEDIATED_PROTEOLYSIS | 1.52 | 0.005 |
|  | KEGG_NON_SMALL_CELL_LUNG_CANCER | 1.48 | 0.035 |
|  | KEGG_AMINO_SUGAR_AND_NUCLEOTIDE_SUGAR_METABOLISM | 1.47 | 0.03 |
|  | KEGG_PANTOTHENATE_AND_COA_BIOSYNTHESIS | 1.47 | 0.022 |
|  | KEGG_OOCYTE_MEIOSIS | 1.47 | 0.016 |
|  | KEGG_GLYCOSAMINOGLYCAN_BIOSYNTHESIS_HEPARAN_SULFATE | 1.46 | 0.035 |
| PRAD | KEGG_MTOR_SIGNALING_PATHWAY | 1.44 | 0.037 |
| BLCA | KEGG_PEROXISOME | 2 | 0.002 |
|  | KEGG_PROPANOATE_METABOLISM | 1.99 | 0.005 |
|  | KEGG_TRYPTOPHAN_METABOLISM | 1.94 | 0.003 |
|  | KEGG_CITRATE_CYCLE_TCA_CYCLE | 1.93 | 0 |
|  | KEGG_ALANINE_ASPARTATE_AND_GLUTAMATE_METABOLISM | 1.88 | 0.006 |
|  | KEGG_PPAR_SIGNALING_PATHWAY | 1.86 | 0 |
|  | KEGG_VALINE_LEUCINE_AND_ISOLEUCINE_DEGRADATION | 1.81 | 0.011 |
|  | KEGG_ALPHA_LINOLENIC_ACID_METABOLISM | 1.8 | 0.003 |
|  | KEGG_TERPENOID_BACKBONE_BIOSYNTHESIS | 1.79 | 0.009 |
|  | KEGG_FATTY_ACID_METABOLISM | 1.79 | 0.005 |
|  | KEGG_STEROID_BIOSYNTHESIS | 1.77 | 0.014 |
|  | KEGG_BASAL_TRANSCRIPTION_FACTORS | 1.75 | 0.013 |
|  | KEGG_SPLICEOSOME | 1.73 | 0.036 |
|  | KEGG_PROTEIN_EXPORT | 1.7 | 0.02 |
|  | KEGG_CYSTEINE_AND_METHIONINE_METABOLISM | 1.69 | 0.012 |
|  | KEGG_AMINOACYL_TRNA_BIOSYNTHESIS | 1.67 | 0.044 |
|  | KEGG_HISTIDINE_METABOLISM | 1.64 | 0.032 |
|  | KEGG_BUTANOATE_METABOLISM | 1.64 | 0.022 |
|  | KEGG_SPHINGOLIPID_METABOLISM | 1.64 | 0.031 |
|  | KEGG_GLYCINE_SERINE_AND_THREONINE_METABOLISM | 1.64 | 0.035 |
|  | KEGG_PYRUVATE_METABOLISM | 1.62 | 0.032 |
|  | KEGG_BASE_EXCISION_REPAIR | 1.61 | 0.046 |
|  | KEGG_RNA_DEGRADATION | 1.59 | 0.04 |
|  | KEGG_LIMONENE_AND_PINENE_DEGRADATION | 1.57 | 0.049 |
|  | KEGG_PHENYLALANINE_METABOLISM | 1.55 | 0.036 |
|  | KEGG_SELENOAMINO_ACID_METABOLISM | 1.54 | 0.045 |
|  | KEGG_GLYCOLYSIS_GLUCONEOGENESIS | 1.52 | 0.042 |
| LIHC | KEGG_BASAL_TRANSCRIPTION_FACTORS | 1.65 | 0.007 |
|  | KEGG_DNA_REPLICATION | 1.62 | 0.011 |
|  | KEGG_DRUG_METABOLISM_OTHER_ENZYMES | 1.64 | 0.009 |
|  | KEGG_GLUTATHIONE_METABOLISM | 1.5 | 0.035 |
|  | KEGG_GLYCOSYLPHOSPHATIDYLINOSITOL_GPI_ANCHOR_BIOSYNTHESIS | 1.68 | 0.007 |
|  | KEGG_LYSINE_DEGRADATION | 1.73 | 0.011 |
|  | KEGG_MISMATCH_REPAIR | 1.66 | 0.002 |
|  | KEGG_N_GLYCAN_BIOSYNTHESIS | 1.81 | 0.002 |
|  | KEGG_NUCLEOTIDE_EXCISION_REPAIR | 1.7 | 0.002 |
|  | KEGG_PROTEIN_EXPORT | 1.64 | 0.036 |
|  | KEGG_SELENOAMINO_ACID_METABOLISM | 1.48 | 0.04 |
|  | KEGG_UBIQUITIN_MEDIATED_PROTEOLYSIS | 1.58 | 0.033 |
|  | KEGG_VALINE_LEUCINE_AND_ISOLEUCINE_BIOSYNTHESIS | 1.51 | 0.045 |
| C-SARC | KEGG_SYSTEMIC_LUPUS_ERYTHEMATOSUS | 1.82 | 0 |
|  | KEGG_TERPENOID_BACKBONE_BIOSYNTHESIS | 1.82 | 0.006 |
|  | KEGG_SPHINGOLIPID_METABOLISM | 1.63 | 0.007 |
|  | KEGG_VIRAL_MYOCARDITIS | 1.62 | 0.032 |
|  | KEGG_LEISHMANIA_INFECTION | 1.56 | 0.049 |
|  | KEGG_N_GLYCAN_BIOSYNTHESIS | 1.55 | 0.03 |
|  | KEGG_FC_GAMMA_R_MEDIATED_PHAGOCYTOSIS | 1.49 | 0.035 |
|  | KEGG_BLADDER_CANCER | 1.35 | 0.038 |
| THCA | NA | NA | NA |
| GBM | NA | NA | NA |
| LGG | NA | NA | NA |
| HNSC | NA | NA | NA |
| R-SARC | NA | NA | NA |
| CESC | NA | NA | NA |
| PCPG | NA | NA | NA |
| UCEC | KEGG_NATURAL_KILLER_CELL_MEDIATED_CYTOTOXICITY | 2.08 | 0 |
|  | KEGG_CITRATE_CYCLE_TCA_CYCLE | 2.08 | 0 |
|  | KEGG_T_CELL_RECEPTOR_SIGNALING_PATHWAY | 2.05 | 0 |
|  | KEGG_RNA_DEGRADATION | 2.05 | 0 |
|  | KEGG_SNARE_INTERACTIONS_IN_VESICULAR_TRANSPORT | 2.01 | 0.004 |
|  | KEGG_FRUCTOSE_AND_MANNOSE_METABOLISM | 2 | 0 |
|  | KEGG_PANTOTHENATE_AND_COA_BIOSYNTHESIS | 2 | 0.002 |
|  | KEGG_GLYCOLYSIS_GLUCONEOGENESIS | 1.99 | 0 |
|  | KEGG_AMINO_SUGAR_AND_NUCLEOTIDE_SUGAR_METABOLISM | 1.99 | 0 |
|  | KEGG_VIBRIO_CHOLERAE_INFECTION | 1.98 | 0 |
|  | KEGG_NUCLEOTIDE_EXCISION_REPAIR | 1.98 | 0 |
|  | KEGG_HOMOLOGOUS_RECOMBINATION | 1.97 | 0.006 |
|  | KEGG_ONE_CARBON_POOL_BY_FOLATE | 1.9 | 0.004 |
|  | KEGG_FC_EPSILON_RI_SIGNALING_PATHWAY | 1.88 | 0.004 |
|  | KEGG_CHEMOKINE_SIGNALING_PATHWAY | 1.87 | 0.016 |
|  | KEGG_PROTEIN_EXPORT | 1.86 | 0.01 |
|  | KEGG_INTESTINAL_IMMUNE_NETWORK_FOR_IGA_PRODUCTION | 1.86 | 0.011 |
|  | KEGG_PYRUVATE_METABOLISM | 1.85 | 0 |
|  | KEGG_VALINE_LEUCINE_AND_ISOLEUCINE_BIOSYNTHESIS | 1.84 | 0.004 |
|  | KEGG_DNA_REPLICATION | 1.84 | 0.01 |
|  | KEGG_TERPENOID_BACKBONE_BIOSYNTHESIS | 1.83 | 0.012 |
|  | KEGG_GRAFT_VERSUS_HOST_DISEASE | 1.83 | 0.011 |
|  | KEGG_AMINOACYL_TRNA_BIOSYNTHESIS | 1.82 | 0.008 |
|  | KEGG_EPITHELIAL_CELL_SIGNALING_IN_HELICOBACTER_PYLORI_INFECTION | 1.81 | 0.008 |
|  | KEGG_LEISHMANIA_INFECTION | 1.8 | 0.033 |
|  | KEGG_VEGF_SIGNALING_PATHWAY | 1.8 | 0.002 |
|  | KEGG_MISMATCH_REPAIR | 1.8 | 0.004 |
|  | KEGG_PYRIMIDINE_METABOLISM | 1.8 | 0.02 |
|  | KEGG_HUNTINGTONS_DISEASE | 1.79 | 0.016 |
|  | KEGG_BIOSYNTHESIS_OF_UNSATURATED_FATTY_ACIDS | 1.79 | 0.016 |
|  | KEGG_OXIDATIVE_PHOSPHORYLATION | 1.77 | 0.037 |
|  | KEGG_OOCYTE_MEIOSIS | 1.77 | 0.02 |
|  | KEGG_BASAL_TRANSCRIPTION_FACTORS | 1.76 | 0.019 |
|  | KEGG_APOPTOSIS | 1.76 | 0.015 |
|  | KEGG_ANTIGEN_PROCESSING_AND_PRESENTATION | 1.76 | 0.045 |
|  | KEGG_VALINE_LEUCINE_AND_ISOLEUCINE_DEGRADATION | 1.76 | 0.024 |
|  | KEGG_UBIQUITIN_MEDIATED_PROTEOLYSIS | 1.75 | 0.03 |
|  | KEGG_TYPE_I_DIABETES_MELLITUS | 1.74 | 0.042 |
|  | KEGG_GLYOXYLATE_AND_DICARBOXYLATE_METABOLISM | 1.73 | 0.012 |
|  | KEGG_ALLOGRAFT_REJECTION | 1.73 | 0.037 |
|  | KEGG_ALZHEIMERS_DISEASE | 1.72 | 0.022 |
|  | KEGG_FC_GAMMA_R_MEDIATED_PHAGOCYTOSIS | 1.72 | 0.016 |
|  | KEGG_PROTEASOME | 1.72 | 0.049 |
|  | KEGG_BASE_EXCISION_REPAIR | 1.71 | 0.028 |
|  | KEGG_RENAL_CELL_CARCINOMA | 1.7 | 0.03 |
|  | KEGG_AUTOIMMUNE_THYROID_DISEASE | 1.7 | 0.043 |
|  | KEGG_P53_SIGNALING_PATHWAY | 1.69 | 0.035 |
|  | KEGG_TOLL_LIKE_RECEPTOR_SIGNALING_PATHWAY | 1.69 | 0.035 |
|  | KEGG_B_CELL_RECEPTOR_SIGNALING_PATHWAY | 1.69 | 0.038 |
|  | KEGG_BLADDER_CANCER | 1.68 | 0.017 |
|  | KEGG_AMYOTROPHIC_LATERAL_SCLEROSIS_ALS | 1.68 | 0.004 |
|  | KEGG_PEROXISOME | 1.68 | 0.041 |
|  | KEGG_VASOPRESSIN_REGULATED_WATER_REABSORPTION | 1.67 | 0.029 |
|  | KEGG_VIRAL_MYOCARDITIS | 1.66 | 0.06 |
|  | KEGG_CYTOKINE_CYTOKINE_RECEPTOR_INTERACTION | 1.66 | 0.03 |
|  | KEGG_PROGESTERONE_MEDIATED_OOCYTE_MATURATION | 1.66 | 0.037 |
|  | KEGG_PORPHYRIN_AND_CHLOROPHYLL_METABOLISM | 1.65 | 0.042 |
|  | KEGG_NOD_LIKE_RECEPTOR_SIGNALING_PATHWAY | 1.64 | 0.039 |
|  | KEGG_ARGININE_AND_PROLINE_METABOLISM | 1.63 | 0.035 |
|  | KEGG_GNRH_SIGNALING_PATHWAY | 1.63 | 0.023 |
|  | KEGG_GLYCINE_SERINE_AND_THREONINE_METABOLISM | 1.61 | 0.027 |
|  | KEGG_NEUROTROPHIN_SIGNALING_PATHWAY | 1.57 | 0.06 |
|  | KEGG_ENDOCYTOSIS | 1.57 | 0.038 |
|  | KEGG_PRION_DISEASES | 1.56 | 0.042 |
|  | KEGG_PPAR_SIGNALING_PATHWAY | 1.54 | 0.039 |
|  | KEGG_MTOR_SIGNALING_PATHWAY | 1.53 | 0.043 |
| TGCT | NA | NA | NA |
| ESCA | KEGG_LYSOSOME | 1.79 | 0.006 |
|  | KEGG_OTHER_GLYCAN_DEGRADATION | 1.78 | 0.016 |
|  | KEGG_PANTOTHENATE_AND_COA_BIOSYNTHESIS | 1.78 | 0.012 |
|  | KEGG_PEROXISOME | 1.7 | 0.036 |
|  | KEGG_SPHINGOLIPID_METABOLISM | 1.65 | 0.037 |
|  | KEGG_PRIMARY_BILE_ACID_BIOSYNTHESIS | 1.63 | 0.035 |
|  | KEGG_VALINE_LEUCINE_AND_ISOLEUCINE_DEGRADATION | 1.63 | 0.043 |
|  | KEGG_VIBRIO_CHOLERAE_INFECTION | 1.53 | 0.032 |
|  | KEGG_PPAR_SIGNALING_PATHWAY | 1.5 | 0.026 |
| STAD | KEGG_SPLICEOSOME | 2.08 | 0 |
|  | KEGG_RNA_DEGRADATION | 2.04 | 0.002 |
|  | KEGG_CELL_CYCLE | 2.03 | 0.002 |
|  | KEGG_NUCLEOTIDE_EXCISION_REPAIR | 2.03 | 0.006 |
|  | KEGG_RNA_POLYMERASE | 2.02 | 0.002 |
|  | KEGG_AMINOACYL_TRNA_BIOSYNTHESIS | 2.02 | 0 |
|  | KEGG_DNA_REPLICATION | 1.94 | 0.006 |
|  | KEGG_BASAL_TRANSCRIPTION_FACTORS | 1.89 | 0.006 |
|  | KEGG_PYRIMIDINE_METABOLISM | 1.87 | 0.013 |
|  | KEGG_HOMOLOGOUS_RECOMBINATION | 1.86 | 0.012 |
|  | KEGG_CITRATE_CYCLE_TCA_CYCLE | 1.85 | 0.016 |
|  | KEGG_ONE_CARBON_POOL_BY_FOLATE | 1.84 | 0.012 |
|  | KEGG_P53_SIGNALING_PATHWAY | 1.82 | 0.008 |
|  | KEGG_PYRUVATE_METABOLISM | 1.79 | 0.007 |
|  | KEGG_MISMATCH_REPAIR | 1.79 | 0.032 |
|  | KEGG_HUNTINGTONS_DISEASE | 1.79 | 0.019 |
|  | KEGG_BASE_EXCISION_REPAIR | 1.78 | 0.028 |
|  | KEGG_OOCYTE_MEIOSIS | 1.74 | 0.023 |
|  | KEGG_VALINE_LEUCINE_AND_ISOLEUCINE_BIOSYNTHESIS | 1.74 | 0.02 |
|  | KEGG_VALINE_LEUCINE_AND_ISOLEUCINE_DEGRADATION | 1.72 | 0.024 |
|  | KEGG_NON_HOMOLOGOUS_END_JOINING | 1.71 | 0.019 |
|  | KEGG_LYSINE_DEGRADATION | 1.7 | 0.049 |
|  | KEGG_GLYOXYLATE_AND_DICARBOXYLATE_METABOLISM | 1.68 | 0.033 |
|  | KEGG_ALZHEIMERS_DISEASE | 1.67 | 0.047 |
|  | KEGG_TERPENOID_BACKBONE_BIOSYNTHESIS | 1.64 | 0.042 |
|  | KEGG_PROPANOATE_METABOLISM | 1.62 | 0.035 |
