# Supplementary material for: From tumor mutational burden to characteristic targets analysis: Identifying the predictive biomarkers and natural product interventions in cancer management
Source: Front Nutr. 2022 Sep 20;9:989989. doi: 10.3389/fnut.2022.989989 (PMC9530334; doi:10.3389/fnut.2022.989989)
Supplement: Supplementary file 13 [file Table_7.DOCX]

| Table S7 GSEA pathway analysis with a single gene-APC for the phenotype | | | |
| --- | --- | --- | --- |
| Name | Size | Enrichment score | P value |
| **Lung adenocarcinoma** |  |  |  |
| regulation of intrinsic apoptotic signaling pathway in response to dna damage | 35 | 0.5619878 | 0.008 |
| establishment of tissue polarity | 121 | 0.441619 | 0.005 |
| negative regulation of intrinsic apoptotic signaling pathway in response to dna damage | 29 | 0.5351841 | 0.011 |
| protein insertion into membrane | 70 | 0.5180539 | 0.005 |
| establishment of protein localization to mitochondrial membrane | 50 | 0.5414957 | 0.014 |
| pentose metabolic process | 15 | 0.65539664 | 0.020 |
| rna 5 end processing | 22 | 0.69793236 | 0.009 |
| protein import into mitochondrial matrix | 19 | 0.7174145 | 0.016 |
| chaperone mediated protein complex assembly | 22 | 0.6087065 | 0.047 |
| regulation of cellular amino acid metabolic process | 60 | 0.63015145 | 0.034 |
| regulation of transcription from rna polymerase ii promoter in response to hypoxia | 74 | 0.5409923 | 0.040 |
| negative regulation of exocytosis | 28 | 0.52458966 | 0.028 |
| release of cytochrome c from mitochondria | 52 | 0.47934088 | 0.005 |
| mitochondrial translational termination | 88 | 0.7028614 | 0.030 |
| regulation of atpase activity | 79 | 0.41167784 | 0.003 |
| positive regulation of mitochondrial outer membrane permeabilization involved in apoptotic signaling pathway | 34 | 0.5262264 | 0.012 |
| translational termination | 103 | 0.66409516 | 0.034 |
| virion assembly | 40 | 0.554273 | 0.022 |
| mitochondrial translation | 130 | 0.653027 | 0.032 |
| regulation of mitochondrial outer membrane permeabilization involved in apoptotic signaling pathway | 43 | 0.4955702 | 0.007 |
| response to arsenic containing substance | 30 | 0.49862504 | 0.011 |
| morphogenesis of a polarized epithelium | 142 | 0.39776418 | 0.006 |
| apoptotic mitochondrial changes | 108 | 0.4294257 | 0.013 |
| proteasome | 42 | 0.7148505 | 0.025 |
| cellular response to cadmium ion | 34 | 0.45356014 | 0.025 |
| trna 5 end processing | 15 | 0.75188893 | 0.013 |
| negative regulation of release of cytochrome c from mitochondria | 15 | 0.6002027 | 0.024 |
| regulation of mitochondrial membrane permeability | 68 | 0.45241663 | 0.012 |
| positive regulation of viral process | 90 | 0.41512102 | 0.019 |
| mitochondrial outer membrane permeabilization | 53 | 0.45643017 | 0.017 |
| regulation of intrinsic apoptotic signaling pathway in response to dna damage by p53 class mediator | 16 | 0.58018243 | 0.026 |
| positive regulation of macrophage chemotaxis | 17 | 0.58584434 | 0.048 |
| negative regulation of regulated secretory pathway | 21 | 0.5391058 | 0.036 |
| mitochondrial membrane organization | 114 | 0.4612219 | 0.017 |
| regulation of release of cytochrome c from mitochondria | 41 | 0.47210467 | 0.022 |
| response to misfolded protein | 23 | 0.5160347 | 0.035 |
| modulation by virus of host process | 29 | 0.49282753 | 0.040 |
| deoxyribonucleoside triphosphate metabolic process | 15 | 0.6159373 | 0.043 |
| cell redox homeostasis | 41 | 0.46823895 | 0.048 |
| positive regulation of membrane permeability | 63 | 0.42553958 | 0.023 |
| positive regulation of ubiquitin protein transferase activity | 33 | 0.45340556 | 0.023 |
| cardiac muscle contraction | 75 | 0.38998464 | 0.036 |
| response to cadmium ion | 56 | 0.39142442 | 0.023 |
| de novo protein folding | 39 | 0.49138126 | 0.026 |
| regulation of mitochondrial membrane potential | 63 | 0.40917966 | 0.021 |
| substantia nigra development | 40 | 0.429068 | 0.031 |
| protein insertion into mitochondrial membrane involved in apoptotic signaling pathway | 30 | 0.48072633 | 0.038 |
| amyloid fibril formation | 24 | 0.46972746 | 0.037 |
| negative regulation of canonical wnt signaling pathway | 169 | 0.34436762 | 0.017 |
| regulation of animal organ morphogenesis | 174 | 0.34094986 | 0.034 |
| regulation of ubiquitin protein transferase activity | 52 | 0.4336443 | 0.035 |
| protein localization to mitochondrion | 140 | 0.4119644 | 0.047 |
| regulation of membrane permeability | 81 | 0.39007294 | 0.023 |
| modulation by virus of host cellular process | 18 | 0.53345734 | 0.049 |
| negative regulation of wnt signaling pathway | 201 | 0.3165965 | 0.021 |
| modulation of process of other organism involved in symbiotic interaction | 93 | 0.3407517 | 0.048 |
| negative regulation of intrinsic apoptotic signaling pathway | 88 | 0.36413866 | 0.044 |
| regulation of intrinsic apoptotic signaling pathway | 146 | 0.33716974 | 0.045 |
| **Lung squamous cell carcinoma** | | | |
| regulation of intrinsic apoptotic signaling pathway in response to dna damage | 35 | 0.5619878 | 0.003 |
| protein insertion into membrane | 70 | 0.5180539 | 0.005 |
| negative regulation of intrinsic apoptotic signaling pathway in response to dna damage | 29 | 0.5351841 | 0.003 |
| regulation of cellular amino acid metabolic process | 60 | 0.63015145 | 0.027 |
| establishment of tissue polarity | 121 | 0.441619 | 0.003 |
| protein import into mitochondrial matrix | 19 | 0.7174145 | 0.014 |
| establishment of protein localization to mitochondrial membrane | 50 | 0.5414957 | 0.007 |
| pentose metabolic process | 15 | 0.65539664 | 0.022 |
| proteasome | 42 | 0.7148505 | 0.018 |
| chaperone mediated protein complex assembly | 22 | 0.6087065 | 0.027 |
| mitochondrial translational termination | 88 | 0.7028614 | 0.024 |
| mitochondrial translation | 130 | 0.653027 | 0.031 |
| translational termination | 103 | 0.66409516 | 0.026 |
| regulation of transcription from rna polymerase ii promoter in response to hypoxia | 74 | 0.5409923 | 0.014 |
| apoptotic mitochondrial changes | 108 | 0.4294257 | 0.006 |
| regulation of atpase activity | 79 | 0.41167784 | 0.006 |
| rna 5 end processing | 22 | 0.69793236 | 0.011 |
| release of cytochrome c from mitochondria | 52 | 0.47934088 | 0.009 |
| regulation of mitochondrial outer membrane permeabilization involved in apoptotic signaling pathway | 43 | 0.4955702 | 0.005 |
| negative regulation of exocytosis | 28 | 0.52458966 | 0.013 |
| regulation of cellular amine metabolic process | 73 | 0.53638995 | 0.038 |
| positive regulation of mitochondrial outer membrane permeabilization involved in apoptotic signaling pathway | 34 | 0.5262264 | 0.012 |
| response to arsenic containing substance | 30 | 0.49862504 | 0.013 |
| mitochondrial membrane organization | 114 | 0.4612219 | 0.009 |
| regulation of mitochondrial membrane permeability | 68 | 0.45241663 | 0.005 |
| morphogenesis of a polarized epithelium | 142 | 0.39776418 | 0.006 |
| positive regulation of macrophage chemotaxis | 17 | 0.58584434 | 0.034 |
| mitochondrial outer membrane permeabilization | 53 | 0.45643017 | 0.007 |
| cellular response to cadmium ion | 34 | 0.45356014 | 0.029 |
| response to misfolded protein | 23 | 0.5160347 | 0.024 |
| negative regulation of release of cytochrome c from mitochondria | 15 | 0.6002027 | 0.028 |
| regulation of intrinsic apoptotic signaling pathway in response to dna damage by p53 class mediator | 16 | 0.58018243 | 0.043 |
| trna 5 end processing | 15 | 0.75188893 | 0.011 |
| negative regulation of regulated secretory pathway | 21 | 0.5391058 | 0.033 |
| virion assembly | 40 | 0.554273 | 0.016 |
| positive regulation of membrane permeability | 63 | 0.42553958 | 0.008 |
| protein transmembrane transport | 59 | 0.47199422 | 0.038 |
| modulation by virus of host process | 29 | 0.49282753 | 0.029 |
| regulation of release of cytochrome c from mitochondria | 41 | 0.47210467 | 0.028 |
| purine nucleoside monophosphate biosynthetic process | 21 | 0.57754874 | 0.034 |
| regulation of mitochondrial membrane potential | 63 | 0.40917966 | 0.019 |
| regulation of dna templated transcription in response to stress | 111 | 0.4363102 | 0.037 |
| response to cadmium ion | 56 | 0.39142442 | 0.030 |
| negative regulation of canonical wnt signaling pathway | 169 | 0.34436762 | 0.014 |
| regulation of animal organ morphogenesis | 174 | 0.34094986 | 0.010 |
| deoxyribonucleoside triphosphate metabolic process | 15 | 0.6159373 | 0.049 |
| protein insertion into mitochondrial membrane involved in apoptotic signaling pathway | 30 | 0.48072633 | 0.036 |
| positive regulation of ubiquitin protein transferase activity | 33 | 0.45340556 | 0.027 |
| positive regulation of viral process | 90 | 0.41512102 | 0.012 |
| substantia nigra development | 40 | 0.429068 | 0.027 |
| regulation of membrane permeability | 81 | 0.39007294 | 0.013 |
| purine containing compound salvage | 15 | 0.54881454 | 0.047 |
| regulation of ubiquitin protein transferase activity | 52 | 0.4336443 | 0.036 |
| negative regulation of wnt signaling pathway | 201 | 0.3165965 | 0.015 |
| negative regulation of protein acetylation | 22 | 0.46580797 | 0.047 |
| regulation of nitric oxide synthase activity | 46 | 0.33061883 | 0.049 |
| **Colorectal cancer** | | | |
| response to misfolded protein | 23 | 0.54106545 | 0.002 |
| purine containing compound transmembrane transport | 16 | 0.6572179 | 0.011 |
| short chain fatty acid metabolic process | 16 | 0.67376995 | 0.018 |
| vitamin transport | 38 | 0.51805496 | 0.010 |
| lipoprotein catabolic process | 15 | 0.5868286 | 0.011 |
| carbohydrate derivative transport | 80 | 0.44465595 | 0.009 |
| aromatic amino acid family metabolic process | 30 | 0.5198102 | 0.005 |
| hydrogen peroxide metabolic process | 52 | 0.48086283 | 0.005 |
| urea cycle and metabolism of amino groups | 20 | 0.5797417 | 0.024 |
| regulation of mitochondrial gene expression | 27 | 0.66604877 | 0.029 |
| hydrogen peroxide catabolic process | 29 | 0.5204427 | 0.021 |
| glycosylation and related congenital defects | 25 | 0.55519116 | 0.033 |
| fucosylation | 16 | 0.5601899 | 0.024 |
| tetrapyrrole biosynthetic process | 27 | 0.53736025 | 0.041 |
| cellular aldehyde metabolic process | 61 | 0.45874417 | 0.028 |
| positive regulation of tor signaling | 39 | 0.44002107 | 0.018 |
| peptidyl l cysteine s palmitoylation | 24 | 0.47993797 | 0.021 |
| tetrapyrrole metabolic process | 57 | 0.44311282 | 0.025 |
| selenium micronutrient network | 72 | 0.4127413 | 0.037 |
| purine nucleotide transport | 24 | 0.4645903 | 0.036 |
| protein quality control for misfolded or incompletely synthesized proteins | 27 | 0.4505157 | 0.028 |
| urea cycle and associated pathways | 24 | 0.53152686 | 0.050 |
| vitamin biosynthetic process | 19 | 0.51040834 | 0.048 |
| glycerolipids and glycerophospholipids | 22 | 0.5014044 | 0.043 |
| glycoside metabolic process | 19 | 0.55146545 | 0.050 |
| iron ion transmembrane transport | 18 | 0.49414414 | 0.039 |
| vacuolar acidification | 23 | 0.48766473 | 0.038 |
| very long chain fatty acid metabolic process | 33 | 0.42426446 | 0.048 |
| regulation of icosanoid secretion | 16 | 0.48846745 | 0.045 |
| cellular iron ion homeostasis | 62 | 0.38624793 | 0.048 |
| detoxification | 117 | 0.35907018 | 0.046 |
