# Supplementary material for: From tumor mutational burden to characteristic targets analysis: Identifying the predictive biomarkers and natural product interventions in cancer management
Source: Front Nutr. 2022 Sep 20;9:989989. doi: 10.3389/fnut.2022.989989 (PMC9530334; doi:10.3389/fnut.2022.989989)
Supplement: Supplementary file 14 [file Table_8.DOC]

| Table S8 Docking results of APC with natural products | | | |
| --- | --- | --- | --- |
| No | Score | Name | structure |
| ZINC4262460 | 14.1612 | Fumonisin B2 | 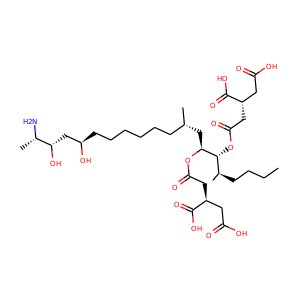 |
| ZINC72170697 | 13.0584 | Fumonisin B1 | 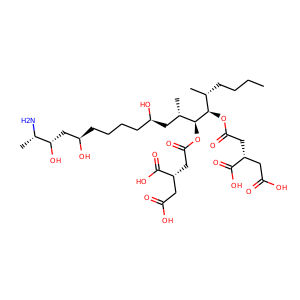 |
| ZINC1542002 | 10.9931 | Fingolimod | 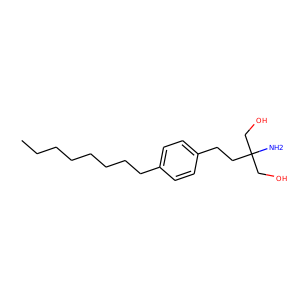 |
| ZINC96903078 | 10.5636 | Jatamanvaltrate B | 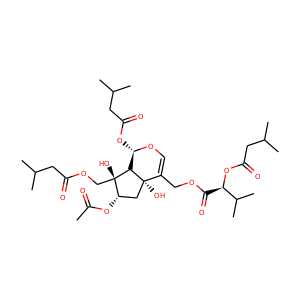 |
| ZINC3830635 | 10.5131 | Dfo | 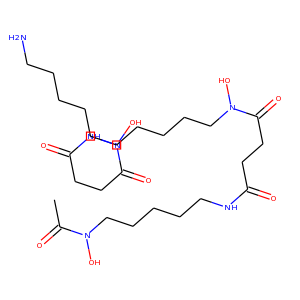 |
| ZINC44387426 | 10.4098 | Isogemichalcone C | 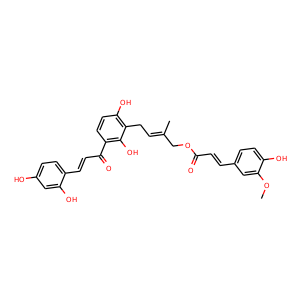 |
| ZINC4475339 | 10.3415 | Phosphoramidon | 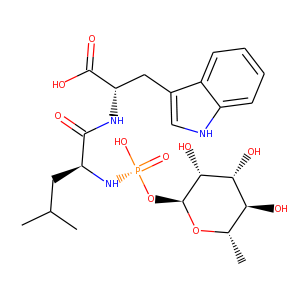 |
| ZINC15253951 | 10.3054 | Cochinchinone B | 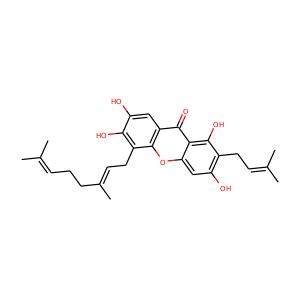 |
| ZINC95542854 | 10.2457 | Lactucain C | 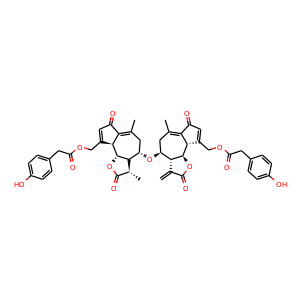 |
| ZINC1530788 | 10.2029 | Cromolyn | 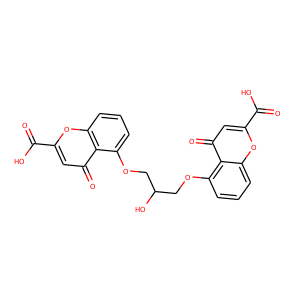 |
| ZINC49872400 | 10.1908 | Piperaduncin C | 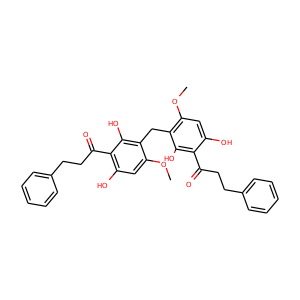 |
| ZINC3874886 | 10.0777 | Rottlerin | 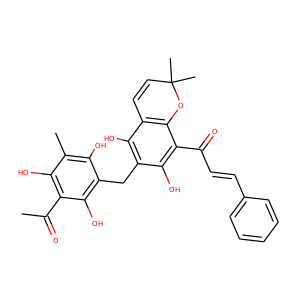 |
| ZINC5782527 | 10.0744 | Myriocin | 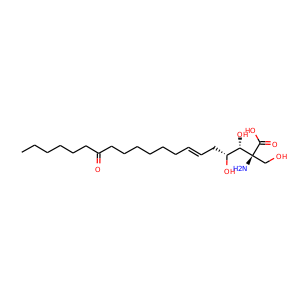 |
| ZINC4098336 | 10.0683 | Didrovaltrate | 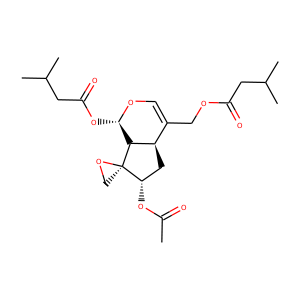 |
| ZINC96903077 | 10.0325 | Jatamanvaltrate B | 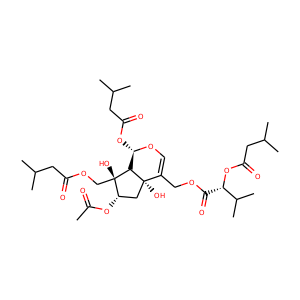 |
| ZINC58568788 | 9.9527 | Ankaflavin | 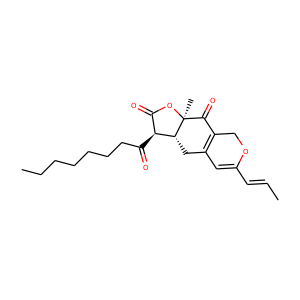 |
| ZINC38944516 | 9.9502 | Cowanol | 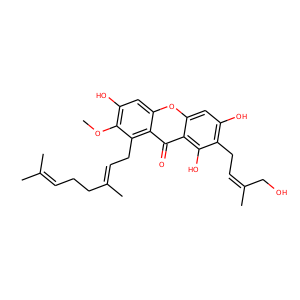 |
| ZINC44306670 | 9.9162 | Cannabisin G | 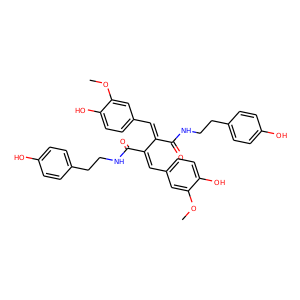 |
| ZINC49850470 | 9.9023 | Manassantin B | 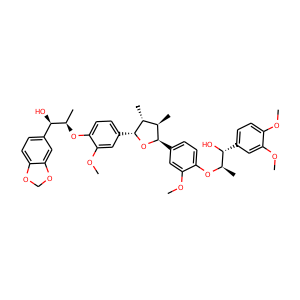 |
| ZINC899362 | 9.8888 | Indicine | 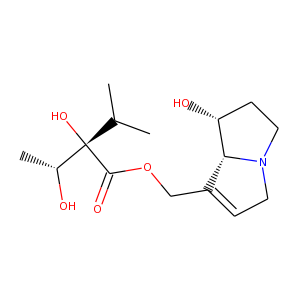 |
| ZINC28968107 | 9.8836 | Cochinchinenin C | 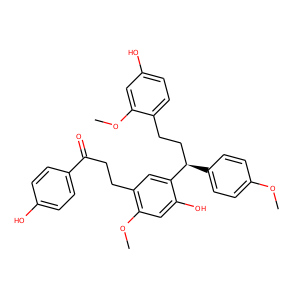 |
| ZINC8214460 | 9.8547 | Sorbitan Monostearate | 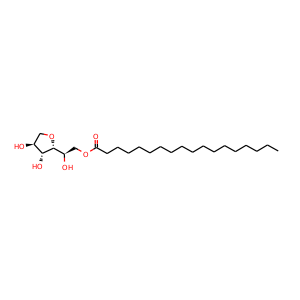 |
| ZINC14233122 | 9.8391 | Fellutamide B | 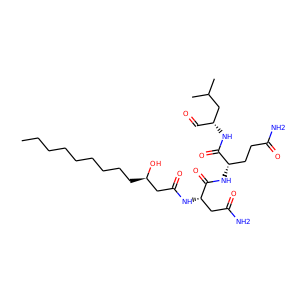 |
| ZINC13377898 | 9.8128 | Hirsutanonol | 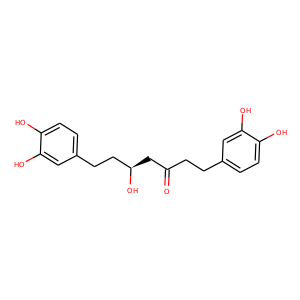 |
| ZINC45337516 | 9.7210 | Gentiside B | 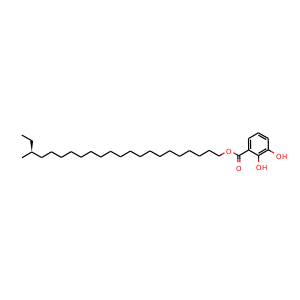 |
| ZINC73220104 | 9.7109 | Docosyl Caffeate | 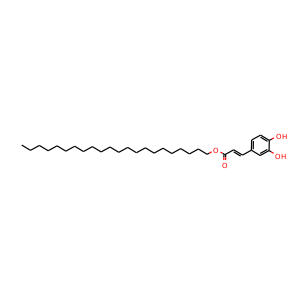 |
| ZINC8214692 | 9.6756 | Tobramycin | 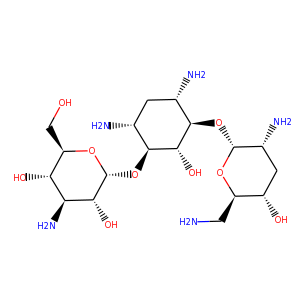 |
| ZINC4098232 | 9.6445 | Phaeantharine | 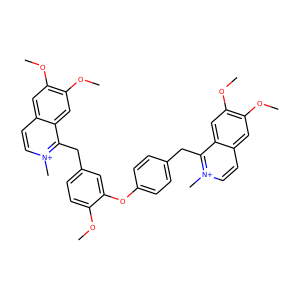 |
| ZINC13377892 | 9.6402 | Octahydrocurcumin | 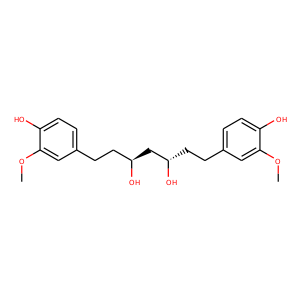 |
| ZINC8214783 | 9.5852 | Phorbol Myristate Acetate | 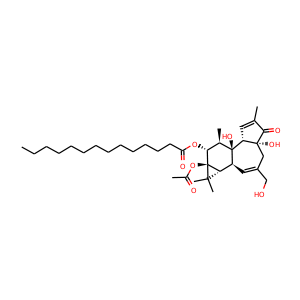 |
| ZINC150354210 | 9.5751 | Oblongifolin C | 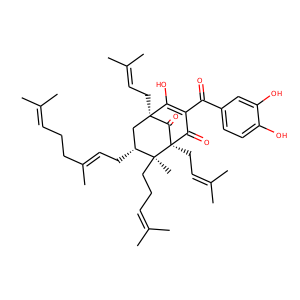 |
| ZINC28968101 | 9.5658 | Cochinchinenin C | 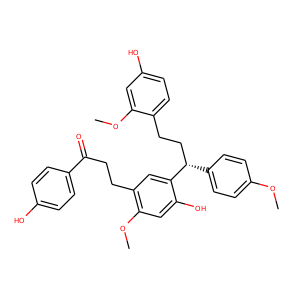 |
| ZINC15266872 | 9.5109 | Haloxyline A | 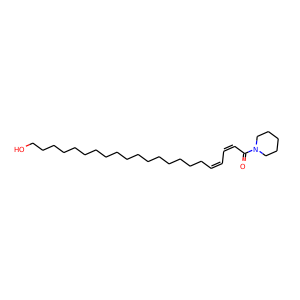 |
| ZINC66065961 | 9.4711 | Tyroscherin 6 | 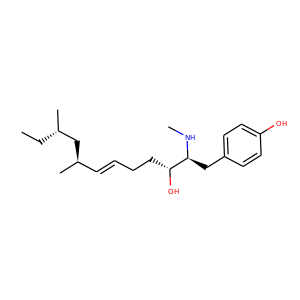 |
| ZINC40863759 | 9.4514 | Macrocarpal D | 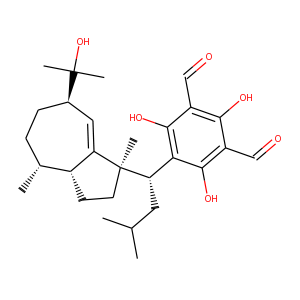 |
| ZINC15120682 | 9.4440 | Cowaxanthone | 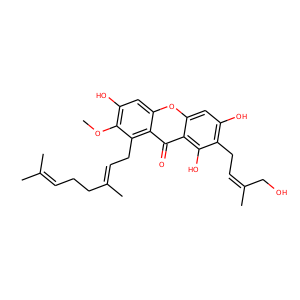 |
| ZINC49888990 | 9.4414 | Epicalyxin I | 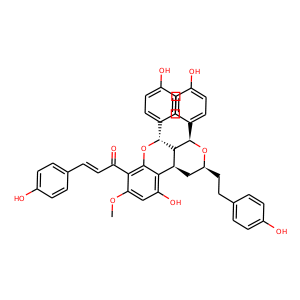 |
| ZINC14718784 | 9.4185 | Surinone A | 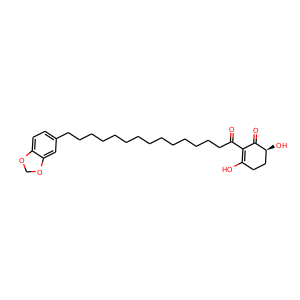 |
| ZINC39066223 | 9.3810 | Kushenol X | 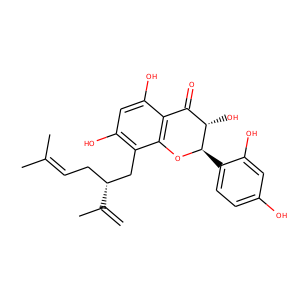 |
| ZINC13377889 | 9.3423 | Hexahydrocurcumin | 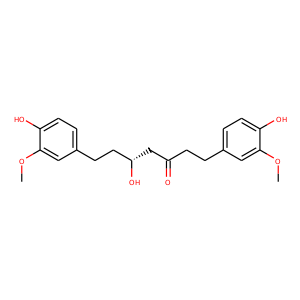 |
| ZINC40976869 | 9.3379 | Tetrahydroxysqualene | 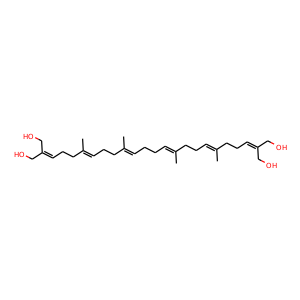 |
| ZINC13606530 | 9.3335 | Harunganol B | 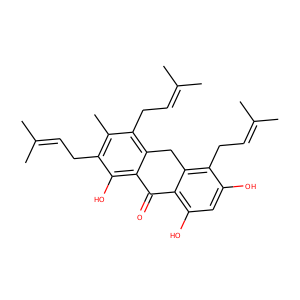 |
| ZINC35458728 | 9.33244 | Tyroscherin 10 | 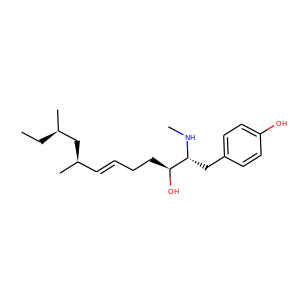 |
| ZINC13377893 | 9.3166 | Octahydrocurcumin | 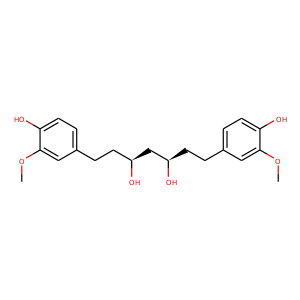 |
| ZINC13373116 | 9.3131 | Arthrobactin | 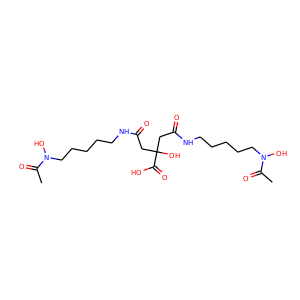 |
| ZINC44430865 | 9.2457 | Hanultarin | 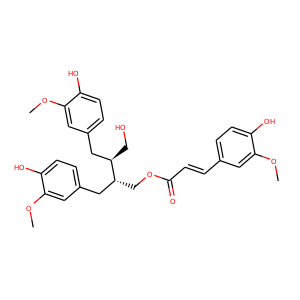 |
| ZINC13377891 | 9.2228 | Octahydrocurcumin | 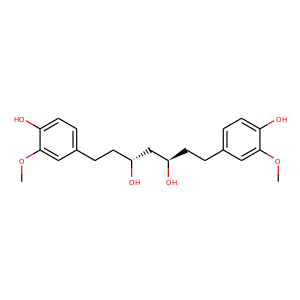 |
| ZINC44307246 | 9.1789 | Lonijaposide A | 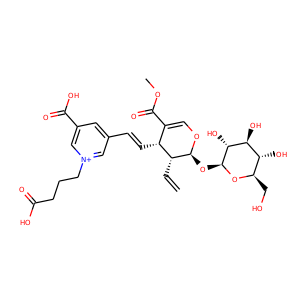 |
| ZINC79212765 | 9.1698 | Pseudojervine | 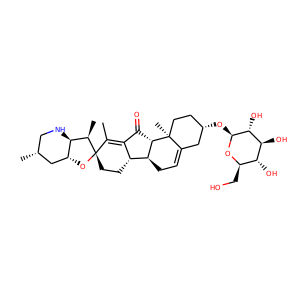 |
| ZINC4102194 | 9.1601 | Centany | 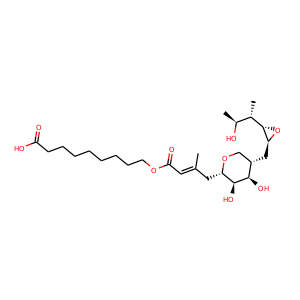 |
| ZINC8143541 | 9.1477 | Gentamicin | 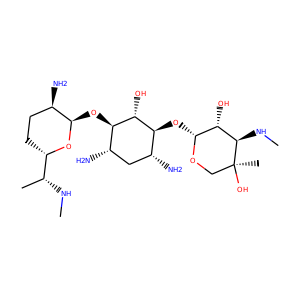 |
| ZINC1564958 | 9.1238 | Malabaricone B | 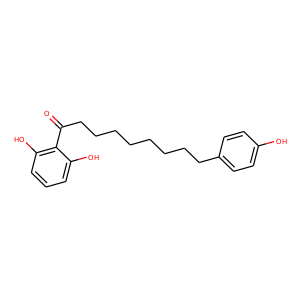 |
| ZINC38336110 | 9.1021 | 1-O-Deisovaeroyl-1-O-3-Methylvaleroyl-Luzonoid A | 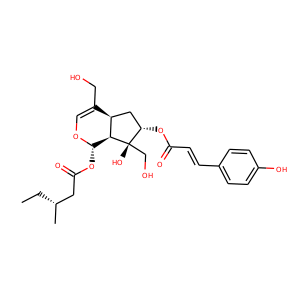 |
| ZINC49933061 | 9.0874 | Dronedarone | 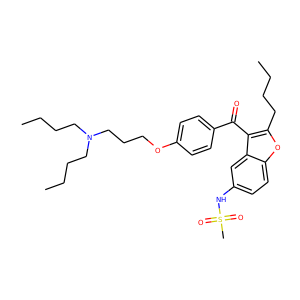 |
| ZINC3871494 | 9.0742 | Alpha-Tochopherol | 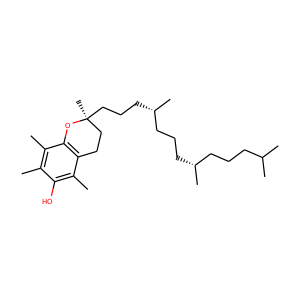 |
| ZINC525679 | 9.0330 | Indicine | 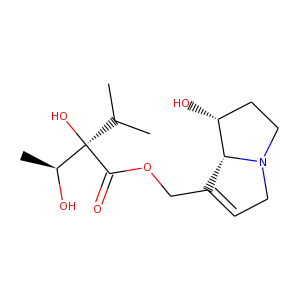 |
| ZINC4098918 | 9.0108 | Phyllanthostatin A | 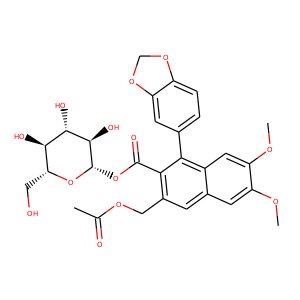 |
| Note: Only results with a score greater than 9 were displayed. | | | |
